# Disruption of the *Caenorhabditis elegans* Integrator complex triggers a non-conventional transcriptional mechanism beyond snRNA genes

**DOI:** 10.1371/journal.pgen.1007981

**Published:** 2019-02-26

**Authors:** Eva Gómez-Orte, Beatriz Sáenz-Narciso, Angelina Zheleva, Begoña Ezcurra, María de Toro, Rosario López, Irene Gastaca, Hilde Nilsen, María P. Sacristán, Ralf Schnabel, Juan Cabello

**Affiliations:** 1 Oncology Area, CIBIR (Center for Biomedical Research of La Rioja), Logroño, La Rioja, Spain; 2 Scientific Computing Group (GRUCACI), University of La Rioja, Logroño, La Rioja, Spain; 3 Department of Clinical Molecular Biology, Institute of Clinical Medicine, University of Oslo and Akershus University Hospital, Lørenskog, Norway; 4 Instituto de Biología Molecular y Celular del Cáncer, Centro de Investigación del Cáncer, CSIC-Universidad de Salamanca, Salamanca, Spain; 5 Department of Developmental Genetics, Institute of Genetics, Technische Universität Braunschweig, Braunschweig, Germany; University of Pennsylvania School of Medicine, UNITED STATES

## Abstract

Gene expression is generally regulated by recruitment of transcription factors and RNA polymerase II (RNAP II) to specific sequences in the gene promoter region. The Integrator complex mediates processing of small nuclear RNAs (snRNAs) as well as the initiation and release of paused RNAP II at specific genes in response to growth factors. Here we show that in *C*. *elegans*, disruption of the Integrator complex leads to transcription of genes located downstream of the snRNA loci via a non-conventional transcription mechanism based on the lack of processing of the snRNAs. RNAP II read-through generates long chimeric RNAs containing snRNA, the intergenic region and the mature mRNA of the downstream gene located *in sense*. These chimeric sn-mRNAs remain as untranslated long non-coding RNAs, in the case of U1- and U2-derived sn-mRNAs, but can be translated to proteins in the case of SL-derived sn-mRNAs. The transcriptional effect caused by disruption of the Integrator complex is not restricted to genes located downstream of the snRNA loci but also affects key regulators of signal transduction such as kinases and phosphatases. Our findings highlight that these transcriptional alterations may be behind the correlation between mutations in the Integrator complex and tumor transformation.

## Author summary

The gene transcription profile determines the developmental state of an organism. During embryogenesis, aging, starvation or any lifecycle stage, organisms express specific sets of genes that must be turned off at other stages to maintain the correct metabolic and differentiated state of the cells. Mutations that disrupt control of signaling pathways may give rise to certain types of tumors. This happens with mutations in genes coding for the Integrator complex, a multi-protein complex involved in the processing of small nuclear RNAs (snRNAs). Here, we uncover a major mechanism underlying gene expression changes in mutants affecting the Integrator complex. Using a *Caenorhabditis elegans* model, we describe how the lack of snRNA processing leads to transcription of genes located downstream of the snRNA loci. This primary alteration is not restricted only to those genes but has a broad effect on the expression of other genes involved in the regulation of signaling pathways by protein phospho-modification.

## Introduction

Transcription is the primary control point for gene expression. It determines cell identity and function and must be finely regulated in each of its steps: initiation, elongation and termination [1,2,3]. Different types of RNA polymerases, as well as other proteins, are involved in these processes. In eukaryotes, RNAP II transcribes protein-coding genes and multiple genes encoding long and small non-coding RNAs. This holoenzyme complex is composed of 12 subunits. The C-terminal domain (CTD) of its largest subunit, Rpb1, plays an essential role in all the transcription regulation steps and couples transcription termination to the processing of nascent RNAs [4,5]. Although the molecular mechanisms of transcription termination are not fully understood, it is widely accepted that 3′-end processing plays a central role. Three different cleavage complexes at the 3’-end have been described, depending on the nascent RNAs: poly(A) mRNAs, replication-dependent histone mRNAs and snRNAs [6]. In the case of snRNAs, the Integrator complex, along with other factors, is responsible for the site-specific cleavage at a conserved sequence named the 3’ box [7,8]. The term “Integrator complex” stands because it integrates the CTD of RNAP II with the 3’-end processing of snRNAs. Initially, 12 subunits were identified and named according to their predicted molecular weight (Integrator subunit, Ints1-12). Proteomic analyses confirmed its composition and identified new putative subunits [9,10]. A genome-wide RNAi screen performed in *Drosophila* S2 cells found two additional subunits that were renamed Ints13 (also known as Asunder) and Ints14 [11]. The Integrator complex is evolutionarily conserved in metazoans. The catalytic subunits Ints11 and Ints9 are clearly homologous to the mammal cleavage and polyadenylation specificity factor subunits, CPSF73 and CPSF100, respectively [12], which are involved in the cleavage of pre-mRNAs and histone mRNAs [13]. Importantly, both belong to a large group of zinc-dependent nucleases called the β-CASP family [14].

Small nuclear RNAs are commonly referred to as “uridine-rich small nuclear RNAs” (U snRNAs) because of their high content in uridine. They are small non-coding RNAs (60–200 nucleotides) that are ubiquitous, intron-less, non-polyadenylated and generally highly expressed. RNAP II transcribes most of the snRNAs (U1, U2, U4, U4atac, U5, U7, U11 and U12), but not U6, which is transcribed by RNAP III [15]. Once the snRNAs are cleaved at their 3’-end by the Integrator complex, they are exported to the cytoplasm for further 3’ trimming and assembled with proteins to form small nuclear ribonucleoproteins (snRNPs) [15,16]. Except for the U7 snRNP that is involved in the 3′-end processing of replication-dependent histone mRNAs [17], snRNPs are components of the spliceosome that mediates pre-mRNA splicing [15], which consists of the removal of introns and ligation of exons within an mRNA molecule [18].

Additionally, in lower eukaryotes such as *C*. *elegans* or *Trypanosoma*, there is another class of snRNAs called spliced leader (SL) snRNAs that are involved in another type of splicing named *trans*-splicing. In SL *trans*-splicing, a short exon is donated from the 5′ end of a SL RNA and connected at or near the 5′ end of an mRNA, thus becoming the first exon of that transcript [19].

Multiple studies implicate some members of the complex in snRNA processing or other biological functions. For instance, Ints3 and Ints6 are involved in the DNA damage response [20,21,22], Ints4 and Ints11 are required for the homeostasis of Cajal bodies [23] and Ints13 is a critical regulator of dynein-mediated processes [24,25,26]. Also, the Ints4, Ints5, Ints6 and Ints7 subunits are essential for normal development in different species [27,28,29,30]. Importantly, recent findings have extended Integrator functions to a broader spectrum of the RNAP II transcription cycle in addition to 3’-end processing, including transcription initiation, promoter-proximal pausing, elongation, and termination [8,31,32,33,34].

Here, we characterize the *C*. *elegans* Integrator complex likely comprised of thirteen subunits (INTS-1, -2, -3, -4, -5, -6, -7, -8, -9, -10, -11, -12 and -13). We show that the Integrator complex is responsible not only for 3’-end processing of snRNAs U1, U2, U4 and U5 as previously described [8,29], but also for SL families and some small nucleolar RNAs (snoRNAs). Strikingly, we observed that depletion of the Integrator complex, results in read-through by the RNAP II leading to transcription of the closest gene located *in sense*. The resulting chimeric RNAs, which we named “sn-mRNAs”, are mostly spliced and polyadenylated and can, in the case of SLs, be translated into proteins. Finally, the transcriptomic profile of Integrator complex depleted nematodes reveals major changes in the kinome and phosphatome as well as other specific genes, pointing to a dramatic alteration of the signaling state of the cells and the existence of other specific functions of the different Integrator subunits.

## Results

### Mutation of *C*. *elegans* Integrator complex subunit 6 causes snRNA misprocessing and embryonic lethality

In a screen for embryonic lethal mutations in *C*. *elegans*, we noticed a striking set of developmental and transcriptional defects in worms and embryos homozygous for the *t1903* mutation. *t1903* was a thermosensitive allele of *dic-1*/INTS6, hereafter referred to as *ints-6* owing to its homology to the human subunit 6 of the Integrator complex INTS6 gene. *ints-6* encodes a protein of 869 amino acids that is highly conserved throughout the animal kingdom. The human homolog has been characterized as a member of the Integrator complex involved in the 3’-end processing of nascent snRNAs [7]. At the molecular level, INTS-6 is predicted to have a von Willebrand factor type A (vWA) domain at the N-terminal part that might serve as a surface for interaction with other proteins or molecules with which it forms complexes [35]. At its C-terminal end it features a COIL domain, which is a structural motif that in many proteins plays a fundamental role in subcellular infrastructure as a molecular ruler, positioning catalytic activities at fixed distances [36].

**Fig 1:**
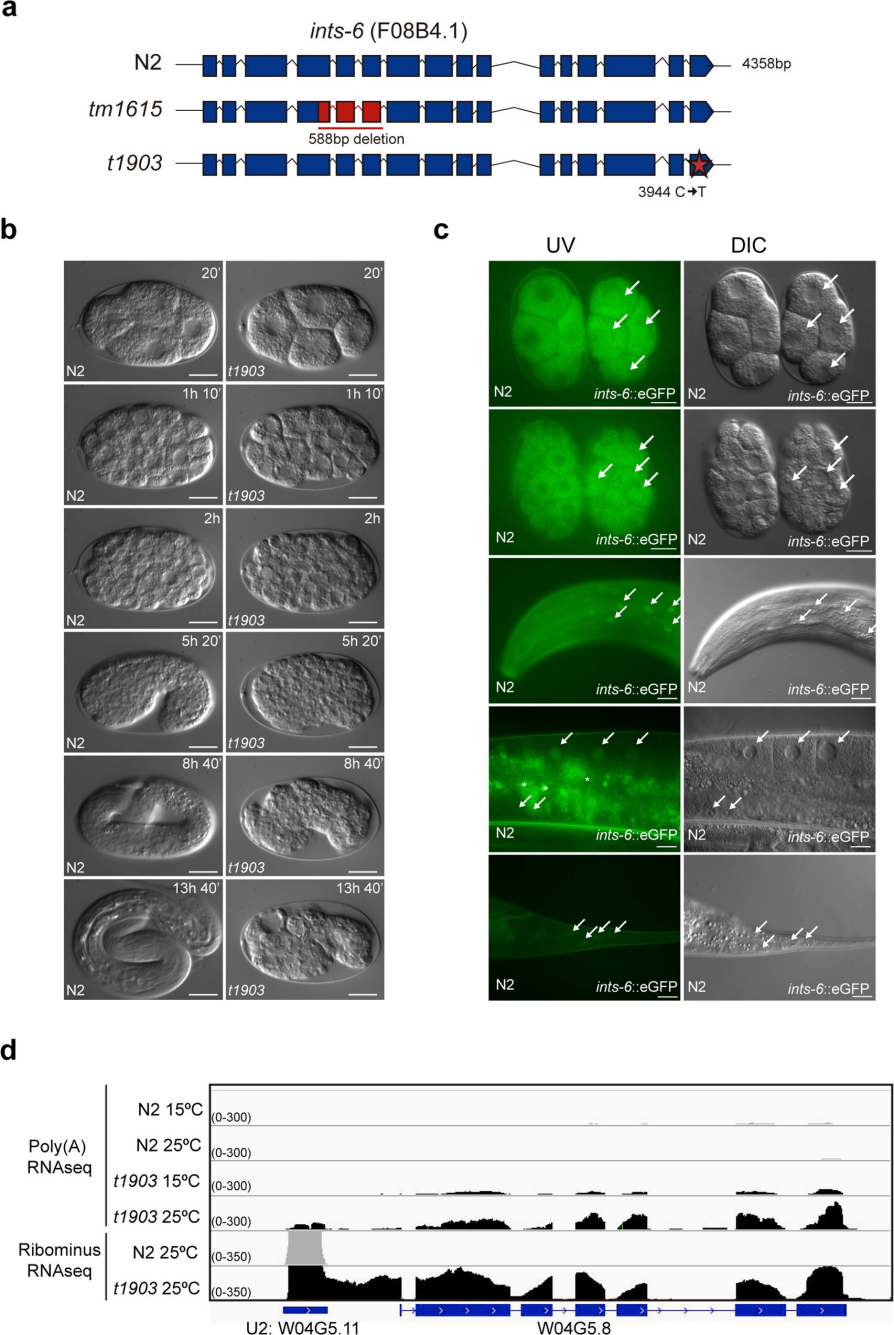
Characterization of *ints-6 (t1903)* mutant. (**a**) Schematic representation of WT *ints-6* gene compared to the knockout *tm1615* and the thermo-sensitive *t1903* mutant (**b**) DIC microscopy images from the two-cell stage show an embryonic lethal phenotype of *ints-6* (*t1903)* whereas WT develops till hatched larvae. Scale bar: 10μm. (**c**) *in vivo* INTS-6 localization in the strain JCP378 (jcpSi19 [pJC56 (*eft-3*p::*ints-6*::3xFLAG::eGFP::*ints-6*UTR, *unc-119*(+))]II; *unc-119(ed3)*III), compared to a N2 WT embryo for autofluorescence (upper two panels). Lower three panels show GFP expression in the head, gonad and tail of the transgenic animals. Arrows indicate a nuclear localization of INTS-6. Scale bar: 20μm. (**d**) RNA deep sequencing reads aligned to the *C*. *elegans* genome in the region of U2 snRNA gene: W04G5.11, visualized on IGV software for each deep sequence technique, RibominusRNA-seq in or Poly(A)RNA-seq. N2 reads are shown in gray whereas *ints-6* (*t1903)* mutant reads are in black. Underneath each graph, the *C*. *elegans* genome is represented in blue. The exons are shown as blue boxes and the introns as lines.

The *t1903* mutation was a C to T substitution at position 3944 in the F08B4.1 gene that resulted in a swap from Ser to Phe in aa 850 of the protein (Fig 1A). At the permissive temperature (15°C), the worms were viable but exhibited some embryonic lethality (21.5%, n = 2153) and reduced offspring (215±23.5 descendats vs 262±20.2 of a WT, mean±sem, n = 10), whereas a shift to the restrictive temperature (25°C) resulted in full embryonic lethality (100%, n = 818) and reduced offspring (82±3.6 descendats vs 185±8.4 of a WT, mean±sem, n = 10) (S1 Fig). A knockout deletion in *ints-6* (*tm1615*) (Fig 1A) [37] has a zygotic effect and homozygotes arrest at the L3 larval stage [38]. To determine the developmental consequences of *ints-6* disruption, we performed four-dimensional (4D) microscope studies on embryos [39]. Analyses of thermosensitive *ints-6* (*t1903*) mutant embryos recorded at the restrictive temperature showed morphogenesis defects leading to embryo death (Fig 1B).

To further address the function of INTS-6, we generated transgenic animals expressing green fluorescent protein-tagged INTS-6 (INTS-6::3xFLAG::eGFP) under the control of its own *ints-6* promoter and the *eft-3* promoter (S2 Fig). Transgenes were assayed both as multicopy extrachromosomal arrays and single copy mosSCI integrated lines. Additional *ints-6*::3xFLAG tagged strains were generated by CRISPR [40,41,42]. Consistent with the localization and function of its human homolog [7,43], the protein appeared as a predominantly nuclear protein (Figs 1C, S3 and S4). Gene expression assessed by GFP detection and by anti-FLAG immunostaining was detected in all cells from embryos to adults. There was, however, a difference between soma and germline expression detected by immunofluorescence: whereas in somatic cells and oocytes INTS-6 was detected in the nucleus, in the embryonic germline it was detected both in the nucleus as well as in cytoplasmic granules (Figs 1C and S4).

To ascertain the role of *ints-6* in 3’-end processing of the snRNAs, as suggested by its homology to the human Integrator complex subunit 6 and its nuclear localization, we performed deep-sequencing of total RNA obtained from *ints-6* (*t1903*) mutant worms. Processing of the 3’ end of snRNAs was already defective at 15°C (permissive temperature) whereas mRNA termination appeared unaffected. Shifting of the *ints-6* (*t1903*) mutant to 25°C (restrictive temperature) for 12h resulted in an increase in the amount of long transcripts derived from unprocessed snRNAs (Figs 1D and S5). 3’-end misprocessing was not restricted to U1, U2, U4 and U5 snRNA [7,29] but also affected SL1 and SL2 snRNAs families and certain small nucleolar RNAs (snoRNAs), but not other types of non-coding RNAs (ncRNAs) such as ribosomal RNAs (rRNAs), transfer RNAs (tRNAs) or lncRNAs (S6 Fig, S1 Table).

These results, homology to human subunit 6 of the Integrator complex, nuclear localization and its role in processing snRNAs, strongly suggest that F08B4.1/*ints-6* is indeed a part of the *C*. *elegans* Integrator complex and prompted us to study the function of this complex in transcription and RNA metabolism during *in vivo* development of a complex organism.

### Identification of the *C*. *elegans* Integrator complex

To define the polypeptide composition of the *C*. *elegans* Integrator complex, we focused on three defining features: its homology to Integrator subunits in other metazoans, the physical association of its subunits in a complex and the lack of 3’-end processing of snRNAs after disruption of the Integrator complex coding genes.

**Fig 2:**
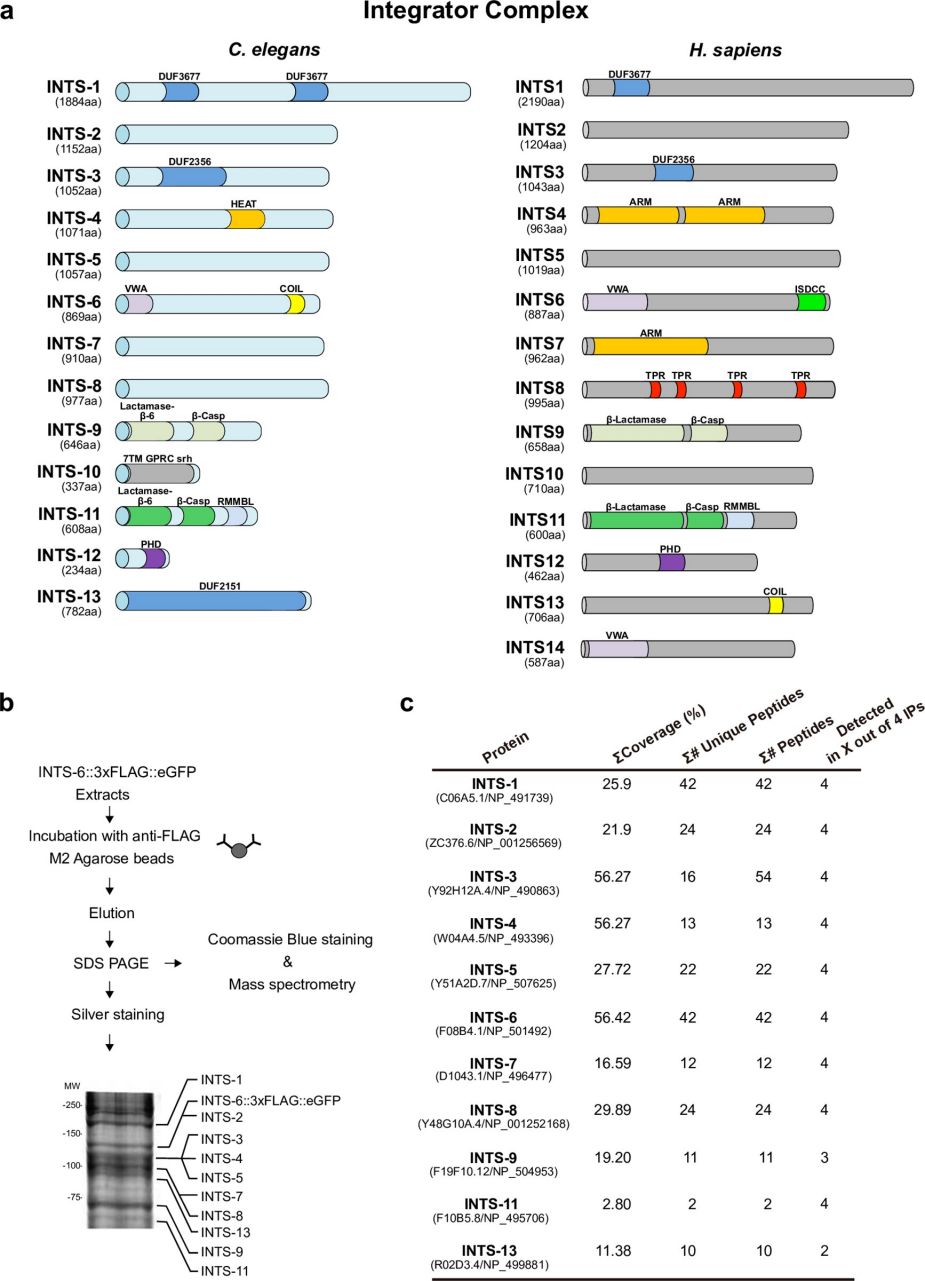
*C*. *elegans* Integrator complex identification. (**a**) Predicted *C*. *elegans* Integrator complex orthologs versus *H*. *sapiens* Integrator complex subunits. Protein domains were searched using the Pfam database. The length of each subunit is indicated in amino acids (aa). Abbreviations: DUF, domain of unknown function; HEAT, Huntingtin, Elongation factor 3, protein phosphatase 2A, and the yeast kinase TOR1; ARM, armadillo-like repeats; COIL, coiled coil domain; VWA, von Willebrand type A domain. β-lactamase/β-CASP (*indicates the presence of an inactive β- lactamase/β-CASP domain); RMMBL, Zn-dependent metallo-hydrolase, RNA specificity domain; 7TM GPCR srh: seven-transmembrane G-protein-coupled receptor, serpentine receptor class h; PHD, plant homeodomain finger; ISDCC, INTS6/SAGE1/DDX26B/CT45 C- terminus; TPR, tetratricopeptide repeats. (**b**) INTS-6 Immunoprecipitation scheme. Protein extracts were obtained from the JCP378 strain. The majority of the immunoprecipitate was analysed by LC-MS/MS. In addition, a small amount of the immunoprecipitate was run on SDS-PAGE and silver stained. The Integrator complex members detected by mass spectrometry are indicated on the silver stained gel according to their expected molecular weight. (**c**) Polypeptides detected from the Integrator complex predicted the orthologs in the four independent INTS-6 co-immunoprecipitation analyses.

A search in the GeneBank database, using the BLAST algorithm, identified protein homologs for the various Integrator subunits in different species (*Homo sapiens*, *Mus musculus*, *Gallus gallus*, *Danio rerio*, *Drosophila melanogaster and C*. *elegans*). Virtually all Integrator subunits except INTS-14 in *C*. *elegans* are conserved throughout evolution (Fig 2A, S2 Table).

Immunoprecipitation followed by mass spectrometry analyses (IP/MS) further confirmed that the proteins identified as *C*. *elegans* Integrator homologs are components of a multiprotein complex. To investigate the polypeptide composition of the *C*. *elegans* Integrator complex, we generated integrated transgenic worms expressing *ints-6*::3xFLAG::eGFP and purified INTS-6-associated proteins by anti-FLAG affinity purification (Figs 2B and S7). WT N2 animals were used as a control for nonspecific binding. The FLAG affinity eluate was separated on a polyacrylamide gel and stained with Coomassie blue dye. Bands of different molecular weight were excised from the gel and subjected to mass spectrometry analysis. This resulted in the identification of INTS-1 (C06A5.1), INTS-2 (ZC376.6), INTS-3 (Y92H12A.4), INTS-4 (W04A4.5), INTS-5 (Y51A2D.7), INTS-6 (F08B4.1), INTS-7 (D1043.1), INTS-8 (Y48G10A.4), INTS-9 (F19F10.12), INTS-11 (F10B5.8) and INTS-13 (R02D3.4) as components of an INTS-6-associated complex. INTS-10 (F47C12.3) and INTS-12 (T23B12.1) subunits were not detected in the mass spectrometry analysis, probably because they are the smallest subunits with molecular weights of 38.7 kDa and 25.9 kDa respectively (Fig 2).

**Fig 3:**
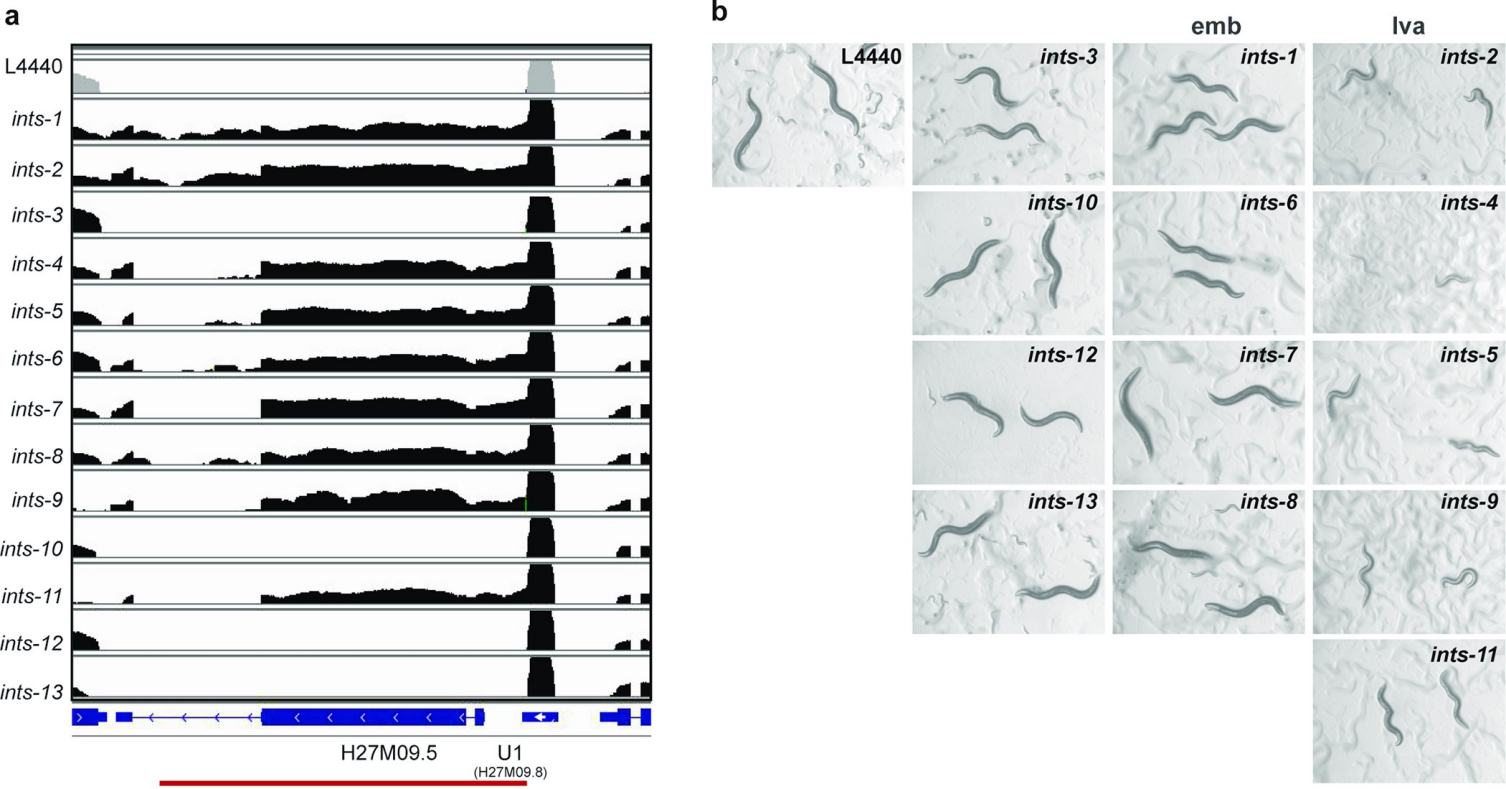
RNAi analysis of the *C*. *elegans* Integrator complex. (**a**) RNA deep sequencing reads aligned to the *C*. *elegans* genome in the region of the U1 snRNA: H27M09.8 and the downstream *in sense* gene H27M09.5 visualized using IGV software for each RNAi Integrator member knockdown (logarithmic scale). L4440 control reads are shown in gray whereas Integrator complex RNAi reads are in black. Underneath each graph, the *C*. *elegans* genome is represented in blue. The exons are shown as blue boxes and the introns as lines. (**b**) Phenotypes of *C*. *elegans* Integrator complex predicted subunits after RNAi knockdown. L1 stage N2 worms were fed bacterial RNAi clones of each predicted member of the Integrator complex. Worms were grown at 15°C. Images were taken on the sixth day (adult stage). *ints-2*, *-4*, *-5*, *-9*, *-11* show a larval arrest phenotype (*lva*), *ints-1*, *-6*, *-7*, *-8* reached adulthood but had reduced offspring that died embryonically (*emb*) whereas *ints-3*, *-10*, *-12*, *-13* showed non-obvious phenotypes.

**Fig 4:**
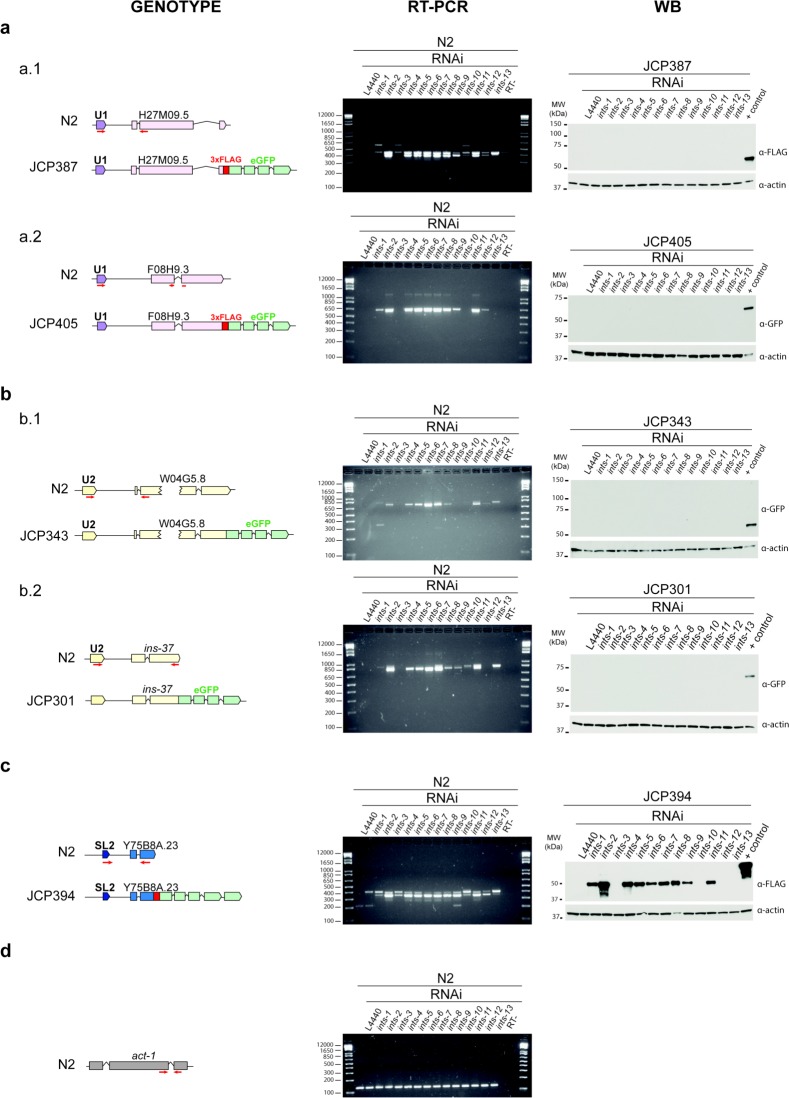
Knockdown of *C*. *elegans* integrator subunits results in formation of long “chimeric sn-mRNAs” that can be translated to proteins in the case of “chimeric SL-mRNAs”. Left panel: schematic representation of the snRNA genes analysed. Red arrows represent the primer pairs used. Middle panel: gel electrophoresis showing RT-PCR analysis. RT minus controls do not show genomic DNA contamination. Double bands correspond to the spliced (lower band) and unspliced (upper band) mRNA fractions. Right panel: Western blot to check the translation of “chimeric sn-mRNAs” into proteins. (**a**) U1 type sn-mRNAs. a.1 PCR expected length: 396 bp (genomic: 439 bp). H27M09.5 estimated molecular weight: 68.8kDa. a.2 PCR expected length: 637 bp. F08H9.3 estimated molecular weight: 46.6 kDa. The U1 derived sn-mRNAs were not translated into proteins (**b**) U2 type snRNAs. b.1 PCR expected length: 782 bp (genomic: 840 bp). W04G5.8 estimated molecular weight: 72.7 kDa b.2 PCR expected length: 804 bp (genomic: 856 bp) INS-37 estimated molecular weight: 45.6 kDa. The U2 derived sn-mRNAs were not translated into proteins (**c**) SL-2 snRNA. PCR expected length: 358 bp (genomic 410bp). Y75B8A.23 estimated molecular weight: 39.5 kDa. The SL derived sn-mRNAs were translated into proteins. (**d**) Actin was used as the loading control: 41.8 kDa. PCR expected length: 156 bp (genomic: 208 bp).

To evaluate the functional contribution of the INTS proteins identified as subunits of the Integrator complex, we assessed 3’-end snRNA processing after RNA interference to knockdown each of them (S8 Fig). 3’-end snRNA processing was determined by RNA deep-sequencing, northern blot and retrotranscription, followed by PCR (RT-PCR) of regions downstream of the snRNA loci (Figs 3A, 4 and S9). U1, U2, U4, U5, SL1, SL2 snRNAs and certain snoRNAs revealed a significant accumulation of long transcripts beyond their 3’ end after depletion of the Integrator subunits INTS-1, -2, -4, -5, -6, -7, -8, -9, -11, whereas other types of ncRNAs, such as rRNAs, tRNAs or lncRNAs remained properly processed at their 3’ end (S1 Table). This indicates a lack of the 3’-end processing of the nascent snRNA transcripts. RNAi knockdown of the Integrator subunits phenocopied the snRNA processing defects observed in the *ints-6* (*t1903*) mutant (Figs 1 and 3) confirming loss of activity of the complex. RNAP II read-through downstream of the snRNAs ranged between 1% and 6% of the total amount of U1 and U2 snRNAs. snRNA expression did not significantly change after depletion of the Integrator subunits and remained as high as in WT (S10 Fig). As a result, read-through transcription downstream of the snRNA loci reached the expression level of regulatory genes such as *lit-1*/NLK or *daf-16*/FOXO of the wnt and insulin pathways respectively (S11 Fig). Depletion of INTS-3, -10, -12 and -13 led only to a slight snRNA misprocessing (Figs 3A and 4). In all cases, mature snRNA transcripts remained at a high level after Integrator depletion (Figs 3 and S9). This is consistent with the reported long half-life of RNAP II-transcribed snRNAs [44]. In addition, stable Integrator complex could retain some activity after RNAi knockdown of single subunits. This explains why mRNA of coding genes is mostly properly spliced, with little intron retention, and polyadenylated after RNAi knockdown of any Integrator complex subunit, as assessed by RNA deep-sequencing and retrotranscription followed by PCR (RT-PCR) of intron-containing regions of coding mRNAs. Interestingly, splicing defects are prominent in the genes located directly downstream of the snRNA loci (15–18% of intron retention for *ints-1*, *-8*, *-9*, *-11* subunit knockdown), whereas genes not affected by RNAP II read-through are only slightly affected (Figs 3A, 4 and S12).

Since the production of ectopic RNAs may have deleterious consequences, we extended the characterization of the phenotypic consequences of Integrator disruption to the level of the whole organism. RNAi of some Integrator subunits exhibited phenotypes ranging from severe larval arrest (*ints-2*, *-4*, *-5*, *-9*, *-11*) to reaching adulthood but having reduced offspring and subsequent embryonic lethality (*ints-1*, *-6*, *-7*, *-8*), whereas the subunits that resulted in only slight snRNA processing defects (*ints-3*, *-10*, *-12*, *-13*), showed non-obvious phenotypes (Fig 3B). These phenotypic differences correlate with the amount of long transcripts detected beyond the 3’ end of the snRNAs after knockdown of the Integrator complex subunits (Fig 3A).

In summary, these findings indicate that the *C*. *elegans* Integrator complex is likely comprised of at least 13 subunits, INTS-1 to INTS-13, involved in the 3’-end processing of snRNAs.

### Integrator disruption causes transcription of genes located downstream of the snRNA loci

To understand the consequences of the lack of 3’-end processing of nascent snRNAs in the organism, we first studied the structure of the uncleaved snRNAs formed upon depletion of any member of the Integrator complex. The structure of the long unprocessed transcripts was assessed by deep-sequencing of total RNA obtained from *ints-6* (*t1903*) and Integrator complex depleted worms (Figs 1D and 3A). This result was further confirmed by northern blot and RT-PCR using specific primers for snRNAs and their downstream genes (Figs 4 and S9). snRNA loci are present in multiple copies within the genome, either in intergenic regions or within coding genes and are oriented either *in sense* or *antisense* to the downstream gene (S1 Table).

For snRNA loci located *in sense* in the 5’ region upstream coding genes, the lack of snRNA processing resulted in the formation of long chimeric sn-mRNAs containing the snRNA sequence at the 5' end, followed by the sequence corresponding to the region between the snRNA and the gene downstream and the mature mRNA of the gene on the 3' end (Figs 1D, 3A and 4). In all these cases, intron retention was detected but most of these transcripts were processed by splicing and polyadenylated at the expected sites (S12 Fig). Thus, depletion of the Integrator complex caused upregulation of genes located downstream of the snRNA loci.

In contrast, for snRNA loci located downstream and opposite to coding genes, the lack of 3’-end processing resulted in the transcription of *cis-antisense* RNAs of the coding genes. Directional deep-sequencing of total RNA from WT and *ints-6* (*t1903*) mutant worms revealed the existence of both types of transcripts: mRNA (*in sense*) derived from the endogenous promoter activity and *antisense* RNAs on the opposite strand, derived from the lack of processing of snRNAs located *in antisense* downstream of the gene. This suggests that these antisense RNAs might not be efficient at performing RNA silencing (S13 Fig). This is consistent with the fact that only double-stranded RNA has been shown to be substantially effective at producing RNA interference. Indeed, injection of purified single strand RNA has at most a modest effect on gene expression [45]. These results reveal the formation of antisense RNAs upon knockdown of the Integrator complex, although its putative function remains an open question.

### Specific long chimeric sn-mRNAs generated upon Integrator disruption are translated into proteins

To establish whether long chimeric sn-mRNAs could be translated to proteins, we generated transgenic worms containing genes that had *in sense* snRNAs in their 5’ upstream region (from 1186 to 213 bp), tagged with FLAG and/or eGFP at the 3’-end of the coding gene in its genomic sequence. The 5 transgenes assayed contained two U1 snRNA genes (H27M09.8 and F08H9.10) in the 5’ upstream region of the tagged genes H27M09.5 and F08H9.3 respectively; two U2 snRNA genes (W04G5.11 and F08G2.9) in the respective 5’ region of the tagged W04G5.8 gene and *ins-37*, an insulin-like peptide; and finally, an SL-2 (*sls-2*.*8*) in the 5’ region of the tagged Y75B8A.23 gene. A single copy of these transgenes was integrated into the *C*. *elegans* genome by mosSCI [41]. Transgenic worms were treated with RNAi of each member of the Integrator complex or crossed with the *ints-6* (*t1903*) mutant. Transgene expression was detected by RT-PCR and protein formation was assessed by western blot (Fig 4).

For the transgenes assayed, we concluded that the lack of 3’-end processing of *sls-2*.*8* snRNA upon depletion of the Integrator complex resulted in transcription of a long chimeric RNA containing *sls-2*, the intergenic region and the downstream gene Y75B8A.23 mostly spliced and polyadenylated. This chimeric RNA was translated into a protein detected by western blot of the FLAG tag at its C-terminal end (Fig 4). Neither the *sls-2* nor the intergenic region contained any ATGs. The first translation start codon that could be used in this long chimeric RNA was the initial ATG of the Y75B8A.23 gene. Y75B8A.23 is a hitherto uncharacterized nematode-specific gene that is highly expressed during spermatogenesis at late larval stages [46].

Lack of 3’-end processing of U1 and U2 snRNAs in the transgenes assayed, caused by RNAi of any Integrator subunit, led to transcription of long chimeric sn-mRNAs. However, these U1- and U2-derived chimeric RNAs were not translated into proteins (Fig 4). We observed that U1 and U2 snRNA genes contain several ATGs in their sequence (S3 Table) as well as a specific secondary structure [47]. To determine whether initial ATGs in these long chimeric sn-mRNAs could serve as start codons for translating peptides, we generated transgenes that contained HA and MYC tags in-frame with the 1^st^ and 2^nd^ ATG of the U1 snRNA gene (F08H9.10) and the U2 snRNA gene (W04G5.11) respectively. Depletion of the Integrator complex by RNAi did not result in peptide formation as determined by western blot using anti-HA and anti-MYC specific antibodies (S14 Fig).

These findings indicate that specific SL-derived, but not U1- or U2-derived, long chimeric sn-mRNAs generated upon Integrator complex downregulation may be translated into proteins.

### Integrator disruption leads to a dramatic alteration of the transcriptomic profile

Downregulation of the Integrator complex has a direct effect on the transcription of genes located downstream of the snRNA loci. To decipher whether gene expression alteration is restricted to those genes or has a broad effect on the general expression profile, we analyzed the long-term transcriptomic profile of nematodes depleted for each member of the Integrator complex by RNAi.

We examined the gene expression profile of WT N2 worms synchronized at the first larval stage (L1) and grown on RNAi feeding plates for each member of the Integrator complex, for 6 days at 15°C. The gene expression data were normalized by a negative binomial distribution model using DESeq2 and EdgeR implementations and compared to a control grown under the same conditions using the empty L4440 vector as the RNAi clone. Three biological replicas of each analysis were performed. Raw sequence data generated in this study are available in the Gene Expression Omnibus (GEO) data repository (Accession number GSE111083). Quantitative analysis of differential expression was performed as described in materials and methods [48].

**Fig 5:**
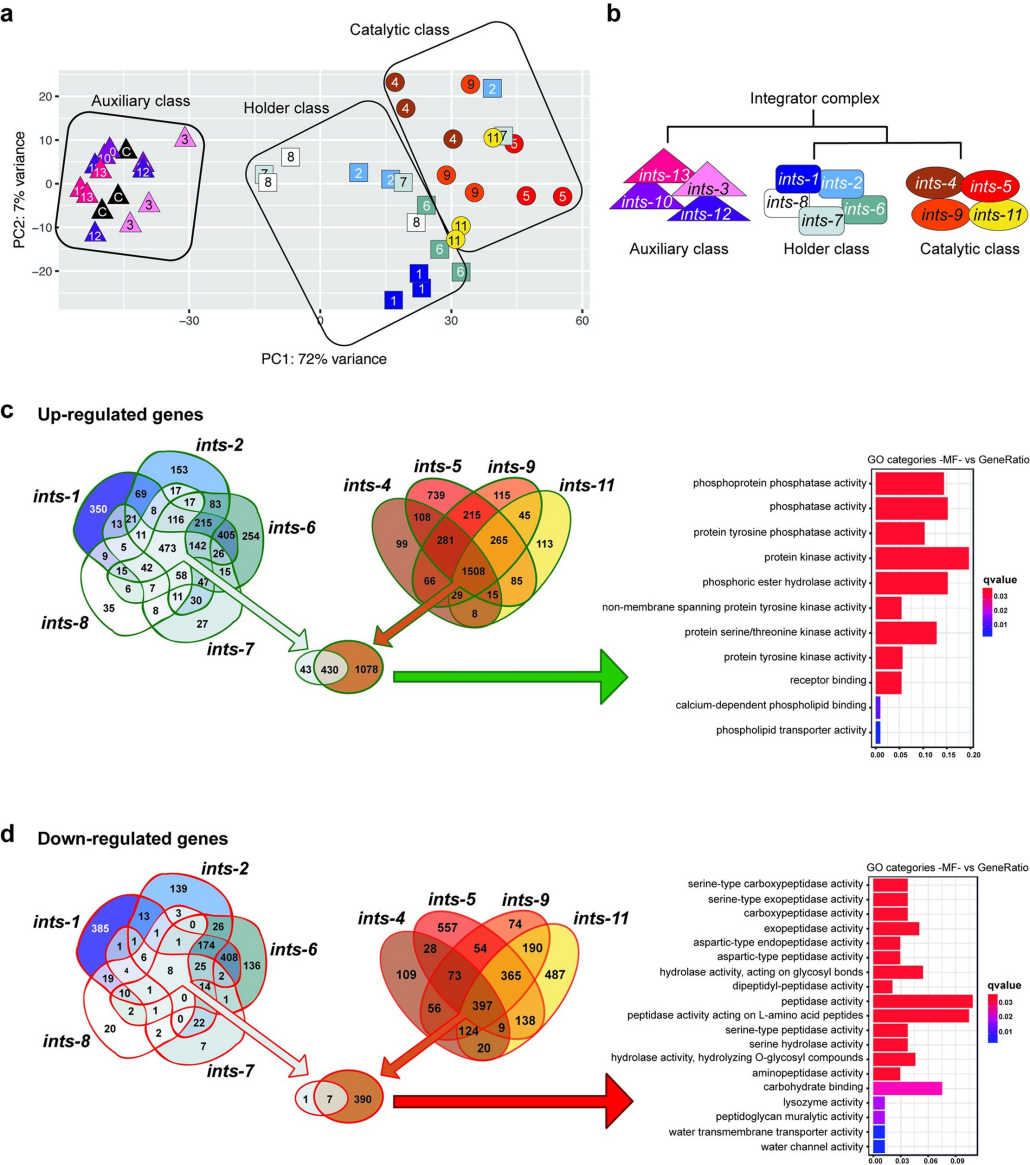
Transcriptomic analysis of the *C*. *elegans* Integrator complex. (**a**) Principal Component Analysis (PCA) plot based on DESeq2 regularized log2 transformation (rld) data shows functional clusters according to the similarity of the transcriptional profile. (**b**) Simplified diagram of Integrator complex members classified as Auxiliary Class, Holder Class or Catalytic Class based on the complete heatmap of the expression profile similarity shown in S15 Fig. (**c**) Venn diagrams of the Holder Class and Catalytic Class showing up-regulated genes. The gene ontology (GO) analysis of upregulated genes’ molecular functions (MF) is shown on the right. GO of MF vs number of genes within each category is shown as color bars, one bar per GO term. Bar length indicates the number of genes belonging to the different GO categories and color illustrates the statistical significance, from those with highly significant expression differences (red) to those with low expression differences (blue). GO functions are highly enriched in kinases and phosphatases. (**d**) Venn diagrams of the Holder and Catalytic Classes showing down-regulated genes. GO analysis of downregulated genes’ MF is shown on the right. GO of MF vs number of genes within each category is shown as color bars, one bar per GO term. Bar length indicates the number of genes belonging to the different GO categories and color illustrates the statistical significance, from those with highly significant expression differences (red) to those with low expression difference (blue).

The Euclidean distance analysis of the global expression profile similarity of the three biological replicas of each Integrator subunit knock-down, plus the control, defined three groups (S15 Fig). These transcriptional groups broadly matched the different phenotypes observed for the depletion of each of the Integrator subunits with punctual replica exceptions (Figs 3, 5A–5B and S15). The transcriptomic profile of the RNAi-depleted worms for the catalytic unit homologs, *ints-9* and *ints-11*, grouped together with *ints-4* and *ints-5*. The highly uniform phenotypic and transcriptional response to the absence of any of these subunits suggests that they are functionally related and defines a category within the Integrator complex hereafter referred to as the Catalytic Class. Integrator subunits exhibiting this Catalytic Class transcriptional signature showed the strongest phenotype after depletion of any Integrator subunits. RNAi of *ints-4*, *ints-5*, *ints-9* or *ints-11* led to a strong lack of 3’-end snRNA processing and to larval arrest of the fed worms (Fig 3). Secondly, the transcriptomic profile of *ints-1*, *ints-2*, *ints-6*, *ints-7*, and *ints-8* RNAi shared some common features with the Catalytic Class. However, their short Euclidean distance in the global expression profile similarity analysis grouped them together in a second transcriptional phenocluster that we hereafter refer to as the Holder Class. RNAi of any Holder Class subunits led to a clear lack of 3’-end snRNA processing. *ints-2* RNAi fed worms arrested as larvae, whereas the rest of the Holder Class subunits (*ints-1*, *ints-6*, *ints-7)* reached adulthood but produced dead embryos in the next generation (Fig 3). Finally, far from the Catalytic and Holder Classes, the transcriptomic profiles of *ints-3*, *ints-10-*, *ints-12-*, *ints-13*-depleted worms did not show significant differences from the control and grouped with WT N2 worms fed the RNAi of the empty L4440 vector. This third transcriptional phenocluster, that we named the Auxiliary Class, makes only a mild contribution to 3’-end snRNA processing under the assayed conditions (Fig 4). In addition, Auxiliary Class subunits RNAi did not show an obvious phenotype (Fig 3). This indicates that under standard laboratory conditions it does not play a central role in snRNA processing and its function, under these specific conditions, is accessory.

To gain insight into the transcriptional role of the three Integrator complex phenoclusters, rather than the biochemical organization of the complex, we identified the overlapping set of genes significantly up- and down-regulated after knockdown of the Integrator subunits within each class (S4 Table). As expected, genes located *in sense* downstream of the snRNA loci that are directly affected by the lack of snRNA processing were upregulated both in the Catalytic and Holder Class RNAi groups. However, these genes constituted only a small fraction of the total altered genes. The rest of the upregulated genes do not have any snRNA in their upstream region as visualized in the RNAseq experiments. Therefore their upregulation is not caused by read-through of the RNAP II downstream of the snRNA. (S1, S4 Table).

**Fig 6:**
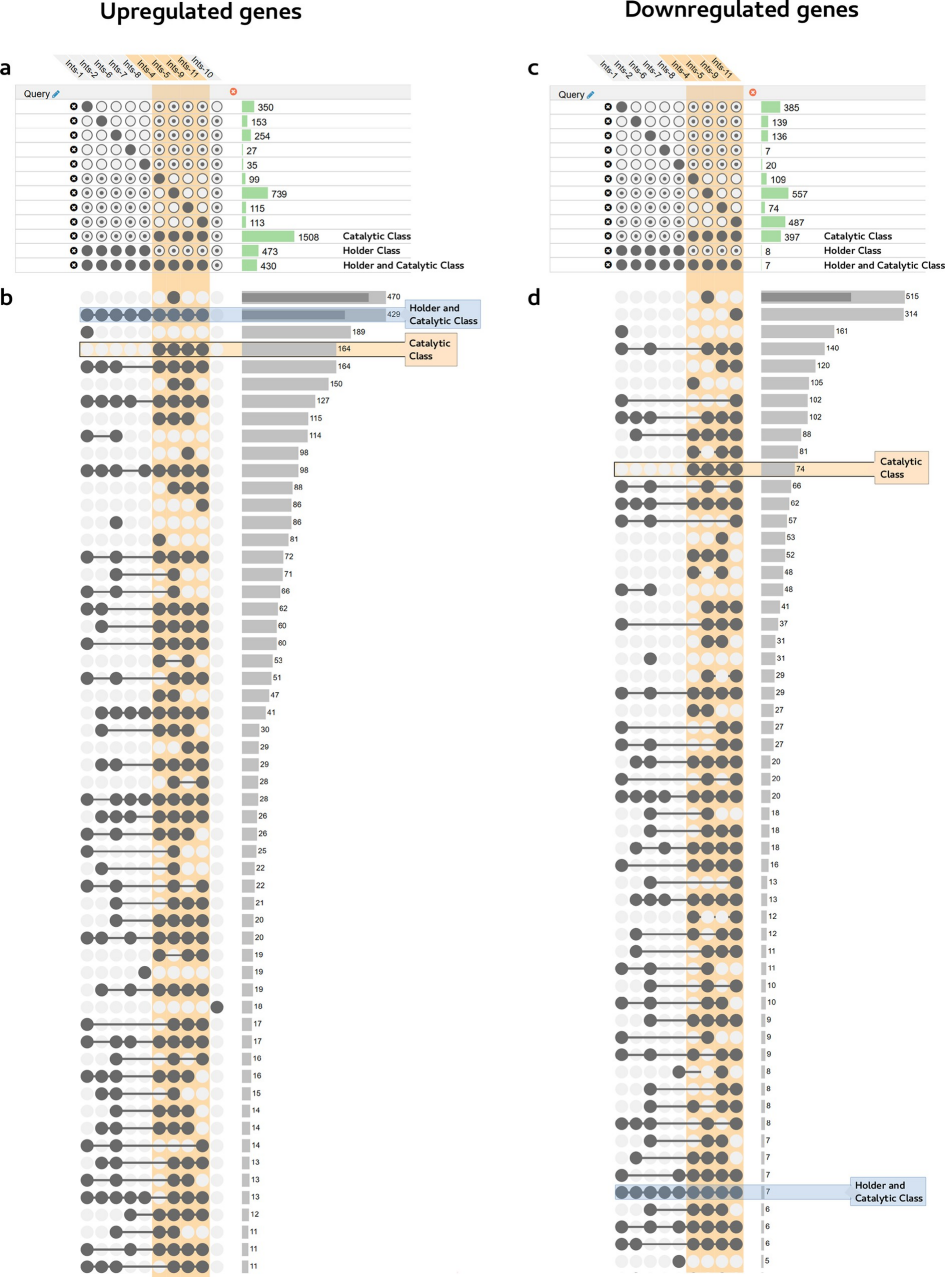
Representation of the up- and downregulated sets of genes in Integrator Complex disruption. This representation was obtained using the Upset web application (www.caleydo.org) and further supports their categorization in different transcriptional classes. Panels show the analysis of upregulated (left) and downregulated (right) genes after depletion of any Integrator subunit. Integrator subunits that significantly affect the expression of a set of genes are shown above. Queries are indicated below as groups of intersections that must (filled circle), may (dotted circle), or must not (empty circle) include a specific set. Histograms on the side represent the number of genes included in each query. Transcriptional catalytic class is highlighted in orange. (a) and (c) show an analysis of genes whose expression is affected by disruption of certain Integrator subunits but may also be affected by others. Therefore, they are present in specific groups of intersections but may also be present in others. (b) and (d) show a restrictive analysis of genes whose expression is specifically affected by disruption of certain Integrator subunits but not by others. Therefore, they are exclusively present in specific groups of intersections and not found in any other sets. Similar to the results shown in Fig 5, Holder and Catalytic classes (blue box) share an upregulated transcriptional signature. In addition to this set of upregulated genes, subunits grouped as Catalytic class (orange box) further show a closer transcriptional signature that affects the upregulation of a specific set of genes absent from the Holder class. In contrast to the level of shared upregulated genes, downregulation does not show such a clear transcriptional signature as indicated by the lower number of shared genes downregulated in the Holder and Catalytic classes. Among the individual Integrator subunits, *ints-1* and *ints-5* show a specific transcriptional signature, both in up- and down-regulated genes, indicating functions in the regulation of specific transcriptional programs. Access to the online data is described in Materials and Methods.

In the Catalytic Class, common genes upregulated by knockdown of any subunit constituted, by far, the largest group of upregulated genes (1508 genes). Upregulated genes specific for the knockdown of any single subunit of the Catalytic Class represented less than 10% of the common response of the class; except for *ints-5* that represented near 50% (Figs 5C and 6) and were enriched in hydrolase, lyase and chitinase activity suggesting a high catabolism rate (S5 Table). This transcriptomic signature highlights a major common function of these subunits. Genes upregulated after depletion of the Integrator Catalytic Class do not randomly fall within different GO molecular function categories. Instead, they are highly enriched in kinases and phosphatases involved in biological processes such as regulation of cell shape, cell proliferation, morphogenesis or signaling pathways. Interestingly, we did not detect activation of stress response pathways (S4 Table). The activation of these pathways depends on ATR kinase that senses blocked transcription elongation rather than DNA lesions directly. In fact, transcriptional and post-transcriptional activation of the stress response occurs when transcription elongation is blocked even in the absence of DNA damage [49,50]. This result strongly indicates that knockdown of the Integrator complex does not abrogate gene transcription. In global terms, upon depletion of the Integrator Catalytic Class, 23% of the total 438 *C*. *elegans* kinases are significantly upregulated (Representation Factor RF = 2.7 p<6.612e-21). Similarly, 38% of the total 206 *C*. *elegans* phosphatases are significantly upregulated (RF = 4.4 p<1.99e-31) (wormbook.org, nemates.org) (Fig 5C). These transcriptomic changes either cause or reflect the dramatic alteration of the signaling state of the cells upon lack of snRNA processing. In contrast to this gene upregulation response, gene downregulation does not show such a clear, common pattern within the Catalytic Class. The set of common genes downregulated by knockdown of any subunit of the Catalytic Class (397 genes) is smaller than the number of genes specifically downregulated by knockdown of *ints-5* (557 genes) or *ints-11* (487 genes). This common downregulation mainly affects peptidases involved in metabolism (Figs 5D and 6).

Regarding the Holder Class, the common genes upregulated by knocking down any subunit (473 genes) overlap in 90% with the Catalytic Class, indicating the involvement of this class in the major activity of the Integrator complex (Fig 5C). However, there is no significant common downregulation response to knockdown of the Holder Class. Among the members of this class, *ints-1* shows a specific effect on the transcription of a subset of genes enriched in extracellular protein coding genes (Figs 5D and 6). Finally, RNAi depletion of Integrator subunits, grouped together as the Auxiliary Class, do not show significant differences from WT under laboratory conditions (S15 Fig).

In addition to the major functions of the Integrator classes, and specific subunits such as *ints-1* and *ints-5*, certain pairs such as *ints-1* and *ints-6* share a fraction of up- and downregulated genes, suggesting a functional relationship between them (Figs 5C–5D and 6). Both sets are enriched in extracellular protein coding genes, indicating a specific function of these subunits in regulating extracellular matrix components. Moreover, although affecting different sets of genes, knockdown of Integrator subunits 1 or 11 causes downregulation of neuronal genes. Knockdown of Integrator subunits 6 and 9 causes downregulation of genes involved in mitochondrial activity. And finally, knockdown of Integrator subunits 7 and 8 causes downregulation of genes coding for gap junction structures (S5 Table). These data are available online for comparison and easy visualization of any of the multiple datasets by loading them onto the web version of the Upset application (http://caleydo.org/tools/upset/) (See Materials and Methods) (Fig 6).

Together, these data indicate that the Integrator complex has a major positive role in processing snRNAs and a negative function in the expression of genes involved in the regulation of signaling pathways by protein phospho-modification. Similar to what occurs in *Drosophila* and mammals, this effect likely reflects the direct role of the *C*. *elegans* Integrator complex on gene expression by regulating the RNAP II gene transcription cycle [8,31,32,33,34]. Additionally, formation of long, unprocessed sn-derived RNAs upon Integrator complex knockdown might have a cascade effect on the expression of other genes.

## Discussion

The global gene expression profile reflects, among other elements, the activity of RNA polymerase complexes on specific genes. This activity is tightly regulated by interaction with transcription factors and other protein complexes such as the Integrator complex. In this work we display the composition of the *C*. *elegans* Integrator as an evolutionarily conserved complex likely composed of 13 subunits: INTS-1 to INTS-13. The existence of similar phenotypes and transcriptomic readouts on some Integrator subunit knockdowns led us to define three different transcriptional clusters: the Catalytic Class (INTS-4, INTS-5, INTS-9, INTS-11), the Holder Class (INTS-1, INTS-2, INTS-6, INTS-7, INTS-8) and the Auxiliary Class (INTS-3, INTS-10, INTS-12, INTS-13).

The transcriptomic profiles of the Integrator subunits are highly homogeneous within the different classes as well as the different replicas. Deviations such as a replica of *ints-2* and *ints-7* (Fig 5A) may reflect the biological and technical variability inherent to experiments with biological samples. Both factors may affect bioinformatic categorizations. In our assay, RNA interference of the Integrator subunits was efficient as indicated by western blot analyses, detection of the RNA-dependent RNA polymerase (RdRp) mediated amplification of the gene transcripts subjected to RNAi silencing and the phenocopy of the snRNA processing defects observed in the *ints-6* (*t1903*) mutant (Figs 1, 3 and S8). Therefore, the resulting bioinformatic categorization justly reflects the similar transcriptional signatures of subunits grouped within the same phenocluster and the closer functional relationship between different intra-cluster subunits (such as INTS-1 and INTS-6) and inter-cluster subunits (such as the Catalytic and Holder Classes) under these experimental conditions. This classification provides a reliable framework in which to classify the different transcriptional outputs. The existence of a common transcriptional signature reflects a functional relationship rather than belonging to a biochemical sub-complex that has not been assessed. The Integrator complex mediates 3’-end processing of snRNAs U1, U2, U4, U5, SLs and certain snoRNAs (indicating the existence of different mechanisms of 3’-end processing for snoRNAs) and has global effects on the transcriptomic profile. However, no effect on the processing of other ncRNAs was observed. Although, so far, no comprehensive analyses have been performed on the phenotypical comparison of the different Integrator subunits, a similar organization could be present in other species. Indeed, human and *Drosophila* INTS4, INTS9 and INTS11 subunits biochemically associate in a module responsible for the catalytic activity of the complex. This module is critical for snRNA 3’-end processing and homeostasis of Cajal bodies [23,51]. Knockdowns of human catalytic subunits INTS9 and INTS11 show similar phenotypes [52]. Mutations in human Ints1 and Ints8, grouped here within the Holder class, cause similar rare recessive human neurodevelopmental syndromes [53]. Finally, in addition to their function in the complex, human INTS10, INTS13 (grouped here within the Auxiliary class), and INTS14 also form a separate module that may be recruited to specific genomic sites to regulate gene expression during monocytic differentiation [54]. In contrast, in humans, INTS3 and INTS6, which in our study show a different transcriptional output, mediate the DNA damage response and form a stable complex even in the absence of DNA damage [22]. Further studies are required to unravel the function of *C*. *elegans* INTS-3 and INTS-6 in the DNA damage response. Certain evolutionary divergence may exist within the Integrator complex, but altogether the data suggest that, bridging the gap between different species, a similar functional output of the different Integrator transcriptional classes might be generally conserved throughout evolution.

Knockdown of the Integrator complex leads to transcription of genes located downstream of the snRNA loci by abrogating the 3’-end processing of the nascent snRNAs. Two scenarios are possible: the gene located downstream of the snRNAs can be orientated either *in sense* or *antisense*. In the first case, the lack of 3’-end processing of snRNAs leads to generation of chimeric sn-mRNAs that are capped, contain the snRNA at the 5’ end of the sequence, continue with the intergenic region and have the mRNA sequence of the downstream gene. In all these cases, intron retention was detected but most of these transcripts were spliced and polyadenylated at the 3’-end (S12 Fig). Transcription in those RNAs ends at the polyadenylation signal of the gene. The opposite orientation of the gene leads to the transcription of chimeric RNAs that possess the snRNA sequence at the 5’ end and an antisense RNA of the downstream gene at the 3’-end.

Our experiments with chimeric sn-mRNAs whose tagged coding mRNA is *in sense* to the snRNA revealed that the assayed U1 or U2 derived sn-mRNAs are not translated into either the protein coded by the gene or peptides starting from previous ATGs within the chimeric sn-mRNAs. Interestingly, SL derived sn-mRNAs can be translated into proteins. This mechanism is different from trans-splicing in which SLs are transcribed, processed to their mature form and fused to the 5’-end of mRNAs from far genomic regions that may even be located on different chromosomes [19]. In the assayed case, disruption of the Integrator complex results in the lack of 3’-end processing of the *sls-2*.*8*/snRNA, the transcription of the downstream intergenic region and the coding gene named Y75B8A.23. The single intron in this gene is mostly spliced and the transcript is polyadenylated.

Taken together, our results show that the capacity to translate these chimeric sn-mRNAs depends on the nature of the snRNA. U-snRNAs have a specific secondary structure oriented to their function in mRNA splicing while SLs might confer stability and enhance the translation of mRNAs containing an sls’ in their 5’ region [19]. Although only SL derived sn-mRNAs can be translated into proteins or peptides, long chimeric U1- and U2-derived and other chimeric sn-mRNA might have an epigenetic effect on gene regulation. This effect could happen directly upon the genes included in the sn-mRNA, but it could also affect other genes by different means such as sequestering microRNAs or epigenetic regulation [55]. In addition to this function in sn-RNA processing, the Integrator RNAPII-associated complex plays a critical role in synchronous activation of gene expression during metazoan development by regulating transcriptional elongation. It is estimated that half of mammalian genes are regulated by pause and release of RNAPII [31–34]. Consistently, our genome-wide analysis reveals that the transcriptional effect caused by disruption of the Integrator complex is not restricted to genes located downstream of the snRNA loci. Likely due to the direct effect of the Integrator complex on gene transcription regulation and as a consequence of the generation of chimeric sn-mRNAs, Integrator disruption affects the transcription of a wide range of genes located away from the snRNA loci. Thus, knockdown of either the Catalytic or the Holder Class subunits of the Integrator complex causes upregulation of genes coding for a large set of kinases (23% of the total kinome) and phosphatases (38% of the total phosphatome). This suggests the unchaining of a dramatic change in the normal regulatory state of the organism’s signaling pathways. In addition to this general effect, knockdown of individual subunits, such as *ints-1* and *ints-5*, affects specific sets of genes, indicating a particular function in transcriptional regulation of extracellular proteins. Other subunits share effects on specific sets of genes involved in mitochondrial activity (*ints-6* and *ints-9*), gap junction activity (*ints-7* and *ints-8*) or neuronal activity (*ints-1* and *ints-11*). This suggests that specific subunits of the Integrator complex might have additional functions beyond the 3’-end processing of the snRNAs, as occurs in humans [31,56].

Our findings indicate that Integrator complex downregulation in *C*. *elegans* triggers non-conventional transcription of genes located downstream of the snRNA loci, generating long chimeric sn-mRNAs. As a result of this and of the direct role of the Integrator complex in transcriptional regulation [31–34], the transcriptomic profile of key regulators in signaling transduction such as kinases, phosphatases and other specific genes, is altered. In humans, alteration in phosphorylation pathways results in serious outcomes in the form of diseases, especially cancer. Phosphorylation-related mutations are highly enriched as tumor “drivers” [57]. Indeed, the tyrosine kinase family encompasses the greatest number of oncoproteins. Altered phosphorylation of proteins involved in cell cycle, apoptosis or cell adhesion pathways corrupt these mechanisms leading to a strong correlation with cancer. As a consequence, kinases offer an enormous potential as targets for drugs in therapies against cancer [58]. Since the characterization of Ints6, named at that time as DICE1 (deleted in cancer 1), as a tumor suppressor in lung carcinomas [59,60], mutations in the different Integrator complex subunits have been reported as involved in multiple kind of tumors [61]. Recent studies show that the Integrator complex is regulated to control the initiation and release of paused RNAP II at immediate early genes (IEGs) following stimulation with epidermal growth factor (EGF) in HeLa cells [31,56]. This raises the possibility that the Integrator complex could be regulated under specific circumstances to produce these chimeric sn-mRNAs that we have observed and to activate a physiological transcriptional response. In this scenario, human tumors that harbor mutations in the Integrator complex might be constitutively activating a similar anomalous transcriptional program to the one described in our *C*. *elegans* model.

## Materials and methods

### Worm strains and culture conditions

*C*. *elegans* strains were maintained on Nematode Growth Medium (NGM) agar plates seeded with a lawn of *E*. *coli* OP50. Bristol N2 was used as the WT strain. The nematodes were grown on these plates at 15°, 20° or 25°C, depending on the purpose of the experiment. The *ints-6* (*t1903)* thermosensitive mutant was regularly grown at 15°C and shifted to 25°C 12h before the experiments, when required. The following strains were used:

JCP294 *ints-6(t1903)* IV

JCP301 jcpSi3 [pJC50 (*ins-37*p::*ins-37*::eGFP::*ins-37*UTR, *unc-119*(+))] II; *unc-119(ed3)* III

JCP341 jcpSi10 [pJC51 (*ints-6*p::*ints-6*::3xFLAG::eGFP::*ints-6*UTR, *unc-119*(+))] II; *unc-119(ed3)* III

JCP343 jcpSi12 [pJC55 (W04G5.8p::W04G5.8::eGFP::W04G5.8UTR, *unc-119*(+))] II; *unc-119(ed3)* III

JCP378 jcpSi19 [pJC56 (*eft-3*p::*ints-6*::3xFLAG::eGFP::*ints-6*UTR, *unc- 119*(+))]II; *unc-119(ed3)*III

JCP383 *ints-6* (*tm1615*) IV; jcpSi10 [pJC51 (*ints-6*p::*ints-6*::3xFLAG::eGFP::*ints-6*UTR, *unc-119*(+))] II

JCP387 jcpSi24 [pJC57 (H27M09.5p::H27M09.5::3xFLAG::eGFP::H27M09.5UTR, *unc-119*(+))]II; *unc-119(ed3)*III

JCP394 jcpSi31 [pJC58 (Y75B8A.23p::Y75B8A.23::3xFLAG::eGFP::Y75B8A.23UTR, *unc-119*(+))]II; *unc-119(ed3)*III

JCP405 jcpSi37 [pJC60 (F08H9.3p::F08H9.3::3xFLAG::eGFP::F08H9.3UTR, *unc-119*(+)]II; *unc-119(ed3)*III

JCP462 *ints-6*(*jcp1*)[*ints-6*::3xFLAG]

JCP479 jcpSi53 [pJC63 (3-tags-in-3-frames (HA:MYC::TY) in the snRNA coding gene F08H9.10, *unc-119*(+))] II; *unc-119(ed3)* III

JCP504 jcpSi55 [pJC64 (3-tags-in-3-frames (HA::MYC::TY) in the snRNA coding gene W04G5.11, *unc-119*(+))] II; *unc-119(ed3)* III

JCP590 *ints-2*(*jcp15*)[*ints-2*::3xFLAG]

JCP614 *ints-3*(*jcp8*)[*ints-3*::3xFLAG]

JCP625 *ints-9*(*jcp10*)[*ints-9*::3xFLAG]

JCP626 *ints-5*(*jcp21*)[*ints-5*::3xFLAG]

JCP630 *ints-7*(*jcp22*)[*ints-7*::3xFLAG]

JCP643 *ints-11*(*jcp31*)[*ints-11*::3xFLAG]

JCP645 *ints-13*(*jcp33*)[*ints-13*::3xFLAG]

### Differential interference contrast microscopy and fluorescence microscopy

Worms were monitored on NGM plates under a Leica dissecting microscope (*MZ16FA*). Gravid hermaphrodites were dissected. 2- to 4-cell stage embryos were mounted on 4% agar pads in water and sealed with Vaseline petroleum jelly. Imaging was performed at 25°C. Differential interference contrast microscopy (DIC) was performed on a motorized fluorescent Leica microscope (*DM6000B*) equipped with a *Hamamatsu Orca-ER C10600* camera and fitted with DIC optics. The appropriate filters were selected for fluorescent microscopy. Images were captured using the open source Micro-manager software (www.micro-manager.org) and processed with XnView software and ImageJ software.

### Embryo and worm immunostaining

Synchronized populations of *C*. *elegans* eggs, larvae or adults were freeze-cracked, fixed with -20°C cold methanol for 2 min and cold acetone for 4 min. Next, samples were dried at RT and recovered by adding a drop of PBS containing 0.1% Tween (PBST) for 5 min. Once eggs or worms were properly prepared, they were blocked for 15 to 30 min in 1% BSA PBST blocking solution. Subsequently, samples were incubated either O/N at 4°C or at RT using FLAG antibody in 1% BSA PBST (F1804 Sigma, 1:500 dilution) followed by 1 or 2 hours incubation with the secondary antibody Alexa Fluor 633 in 1% BSA PBST (Invitrogen A-21050, 1:500 dilution). Finally, samples were mounted using VECTASHIELD Antifade Mounting Medium (H-1000 Vector laboratories) with DAPI (1 μg/ml).

### Immunocytochemistry

U2OS cells were cultured and incubated on pre-treated poly-L-Lysine coated coverslips (Sigma) to improve adhesion. Cells were washed with PBS/Ca^2+^Mg^2+^ (1 mM) and fixed with 4% paraformaldehyde in PBS/Na^+^K^+^ (1 mM) for 30 min at RT with gentle agitation. Next, two washes with PBS (1 mM) were performed for 5 min each and then cells were permeabilized with 0.1% Triton X-100/PBS for 10 min at RT with gentle shaking.

Next, cells were washed with 0.2% PBS-BSA for 10 min at RT. Subsequently, cells were incubated with Ints6 antibody (Bethyl Laboratories, 1:50 dilution) in 0.2% PBS-BSA for 1 h at RT in a humid chamber. Afterwards, cells were washed with PBS (3 times, 7 min each) and incubated with the secondary antibody (*CyTM3-conjugated AffiniPure Goat Anti-Rabbit IgG*, 115-165-003, Jackson ImmunoResearch Laboratories, 1:500 dilution) in 0.2% PBS-BSA for 30 min at RT in the dark. After this incubation, cells were washed with PBS (3 times, 5 min) and incubated with DAPI (2 μg/ml) in PBS for 5 min, all in the dark. Finally, cells were washed with Milli-Q water (3 times) and coverslips were mounted on the slides using the *SlowFade Antifade kit* (Invitrogen).

### Immunoprecipitation

To immunoprecipitate INTS-6, and co-immunoprecipitate its interactors, protein extracts from the JCP378 strain (jcpSi19[pJC56(*eft-3*p::*ints-6*::3xFLAG::eGFP::*ints-6*UTR,*unc-119*(+))]II;*unc-119(ed3)*III) were used and extracts from N2 worms were the negative control. IPs/Co-IPs were performed with *ANTI-FLAG M2 Magnetic Beads* (Sigma).

Protein extracts were filtered through a 5.0 μm filter and subsequently through 0.45 μm filters to remove any remaining cell debris and particulates that could interfere with protein binding. In each IP/Co-IP reaction, 150 μl of the *ANTI-FLAG M2 Magnetic Beads* were incubated with 30 mg of protein extract.

Immediately afterwards, the beads were equilibrated with TBS buffer (375 μl per IP/Co-IP reaction: 50 mM Tris HCl, 150 mM NaCl, pH 7.4). This step was repeated, leaving the beads with a small amount of buffer. Then, the protein extract was incubated with the equilibrated beads for 3–4 h or O/N, always at 4°C in a rotating rack with gentle mixing. Once the binding step was complete, the beads were collected and the supernatants were removed, followed by the washing steps. The beads were washed with TBS buffer (1500 μl per IP/Co-IP reaction) three sequential times for 10 min on a rotating rack at 4°C to remove all non-specifically bound proteins.

INTS-6::3xFLAG::eGFP fusion protein and consequently its interacting proteins were eluted from the magnetic beads either by boiling samples in SDS-PAGE sample buffer (100 μl) or by competitive elution with 3xFLAG peptide (400 ng/μl 3xFLAG peptide in TBS) for 1 h at RT in a rotating rack with gentle mixing). Finally, eluates were precipitated using TCA.

### Mass spectrometry

Eluted IPs were run on SDS-PAGE (*Mini-PROTEAN*™ *TGX*™ *Precast Gels*, Any kDa). Next, gels were stained with Coomassie Blue and bands were excised. The CIC bioGUNE proteomics platform (https://www.cicbiogune.es/org/plataformas/Proteomics) performed the proteomic analysis. Proteins were digested with Trypsin from each gel band and analyzed by LC-MS/MS: Liquid Chromatography-Mass Spectrometry/Mass Spectrometry.

### Protein extraction

Worms (usually from 8 to 10 NGM plates) were harvested with M9 buffer and collected in 50 ml Falcon tubes. They were washed several times, allowing them to settle to the bottom between washes. After the last washing step, as much supernatant as possible was removed. Then, a double volume of lysis buffer (50 mM Tris HCl pH 7.4, 150 mM NaCl, 1 mM EDTA, and 1% Triton X-100), containing 1x protease inhibitors (*Complete EDTA-free Protease inhibitor*, Roche) and 1x phosphatase inhibitors (*PhosSTOP*, Roche) was added to each worm pellet. Next, samples were ground in liquid nitrogen using a pre-chilled mortar and pestle. Ground worms were thawed on ice followed by centrifugation at 4°C (15000 rpm, 15 min) to eliminate any non-soluble tissue or cellular remains. The supernatants were transferred to fresh Eppendorf tubes.

### SDS polyacrylamide gel electrophoresis (SDS-PAGE)

30 μg of protein extract per sample were loaded onto the polyacrylamide gels (*Mini-PROTEAN TGX Precast Gels*) after boiling for 5 min in Laemmli buffer (80 mM Tris-HCl pH 6.8, 5 mM DTT, 2% SDS, 7.5% glycerol, 5 mM EDTA, 0.002% bromophenol blue). Samples were run using the *Mini- PROTEAN Tetra Cell* or the *Criterion™ Cell* electrophoresis system (Bio-Rad) at a constant voltage of 120 V in SDS-PAGE running buffer (25 mM Tris, 200 mM glycine, 0.1% (w/v) SDS) until the tracking dye reached the bottom of the gel. *Precision Plus Protein Dual Color Standards* (Bio-Rad) were used as the size reference.

### Western blot analysis

For antibody-specific detection of proteins, samples were separated by SDS- PAGE and transferred to a 0.45 μm nitrocellulose membrane (*Protran BA 85*, GE Healthcare) in transfer buffer (25 mM Tris, 192 mM glycine, 10% methanol) for 90 min at 4°C and a constant voltage of 90 V using the *Mini Trans-Blot Electrophoretic Transfer Cell* (Bio-Rad) or *Criterion Blotter*. After transfer, the membrane was blocked in TBS-T (49 mM Tris base 102 mM NaCl, 5.4 mM KCl, 0.05% (v/v) Tween-20, pH 8) with 5% (w/v) non-fat dry milk (*Sveltesse* Nestlé) for 60 min with gentle rocking. To detect the protein of interest, the membrane was incubated with the primary antibody (HA 6E2, Cell Signaling 1:1000; GFP Living Colors GFP Monoclonal 632381 Clontech 1:1000; FLAG F1804 Sigma 1:1000; TY1 SAB4800032 Sigma 1:1000; MYC 9B11 Cell Signaling 1:1000; actin (I-19) sc-1616 Santa Cruz Biotechnology 1:1000) diluted in TBS-T milk for 120 min at RT or O/N at 4°C. Afterwards, the membrane was washed three times with TBS-T for 10 min each and then incubated with the respective horseradish peroxidase (HRP) conjugated secondary antibody in TBS-T milk (α-mouse HRP linked GE Healthcare NA931 1:2500; α-goat IgG HRP linked 805-035-180 Jackson ImmunoResearch 1:5000) for 60 min at RT. The membrane was washed twice more with TBS-T for 10 min each time and then once with TBS only.

To chemiluminescently detect the protein of interest, *ECL Blotting Detection Reagents* (GE Healthcare) and *Amersham Hyperfilms ECL* (GE Healthcare) were used according to the manufacturer’s instructions.

Films were developed manually: 1 min in developing solution (Agfa developer G153), 1 min in fixing solution (Agfa fixer G-345) and then rinsed in water.

### Coomassie blue and silver staining

Polyacrylamide gels were stained with Coomassie blue using the *Colloidal Blue Staining Kit* (Invitrogen) according to the manufacturer’s instructions. Polyacrylamide gels were silver stained using Silver Stain for Mass Spectrometry (Thermo Scientific) according to the manufacturer’s instructions.

### RNA interference

Plates seeded with the corresponding RNAi clones were used to feed synchronized WT L1 worms. RNAi clones were obtained from either the ORFeome Library [62]: ZC376.6 (*ints-2*), Y51A2D.7 (*ints-5*) T23B12.1 (*ints-12*) R02D3.4 (*ints-13*) or the Ahringer Library [63]: F47C12.3 (*ints-10*).

The RNAi clones C06A5.1 (*ints-1*), Y92H12A.4 (*ints-3*), W04A4.5 (*ints-4*), F08B4.1 (*ints-6*), D1043.1 (*ints-7*), Y48G10A.4 (*ints-8*), F19F10.12 (*ints-9*), F10B5.8 (*ints-11*) were cloned from cDNA using the following primers:

*ints-1* (Fw 5’-AAACCACGAGTTGGACAAGG-3’,

Rv 5’-TCAAATCAATCGGCATTTCA-3’),

*ints-3* (Fw 5’-TTCGCCAAAATGTGAAACAA-3’,

Rv 5’-AGACGTAGGTCAGCGAGGAA-3’)

*ints-4* (Fw 5’- CGGATCCCAGAAGAATCGTA-3’,

Rv 5’-CGTCATCACTTGCATCATCC-3’)

*ints-6 (*Fw 5’- CTCGTTTGAATCCACAAGCA-3’,

Rv 5’-TGAGCTTTTGAGGCATGTTG-3’)

*ints-7 (*Fw 5’- TGTGAATGCGATGCTTCTTC-3’,

Rv 5’ ACATGTACGGGCAGTTGTCA-3’)

*ints-8 (*Fw: 5’-TTACTAAGCTTCCATAGATCGCCGTAATCGT-3’,

Rv: 5’- TTACTCTCGAGGTGAGTGGGCCGTGAAGTAT-3’),

*ints-9 (*Fw: 5’-TATATCAAAGCCCGCGAATC-3’,

Rv: 5’- GGTCTCATCCGGTTTTCAA-3’)

*ints-11* (Fw: 5’- AAAAAGGTTGTCGGATGTGC-3’,

Rv: 5’- GCTTCGGTTGAGCAGAAATC-3’)

All RNAi clones were verified by sequencing.

5 ml LB medium containing ampicillin (100 μg/ml) was inoculated with a single bacterial colony and incubated at 37°C for 8 h with constant shaking. 400 μl of the bacterial culture was spread on 90 mm NGM RNAi feeding plates (NGM plates with 100 μg/ml ampicillin, 12.5 μg/ml tetracycline, 1 mM IPTG) and incubated O/N at RT to grow a bacterial lawn and induce dsRNA expression. The next day, synchronized L1 populations were transferred to RNAi feeding plates. The WT strain fed with a clone carrying the empty L4440 vector was used as an RNAi negative control. Functioning of RNAi was assessed by detection of RNA-dependent RNA polymerase mediated amplification of the gene transcripts subjected to RNAi silencing, and western blot of representative subunits of the three transcriptional classes (S8 Fig).

### RNA extraction

Worms from 3 to 5 plates were washed off with M9 buffer and collected in 50 ml Falcon tubes, allowing them to settle to the bottom of the tube. Worm pellets were subsequently washed with M9 buffer until no bacterial remains were visible. Next, worm pellets were transferred to Eppendorf tubes and as much supernatant as possible was removed. RNA extractions were performed using the *mirVana™miRNA isolation kit* (Ambion) following the manufacturer’s protocol for total RNA isolation. Worm tissue homogenization was carried out with the assistance of a polytron pre-chilled with liquid nitrogen.

### Northern blot

7 μg of total RNA was denatured for 5 minutes at 95°C and loaded onto denaturing 10% polyacrylamide gels containing TBE-Urea (Bio-rad). Electrophoresis run for about 150 min at 150 V. RNA was transferred to positively charged nylon membranes (Hybond-N+, Roche). After brief washing using 2 × SSC, the transferred blots were cross-linked under short-wave UV light. After prehybridization at 50°C for 1 hour in Church Buffer (0.36M Na2HPO4, 0,14M NaH2PO4, 7% SDS, 1mM EDTA), the blots were subjected to hybridization with Digoxigenin-labeled DNA probes overnight at 50°C. The membranes were washed as follows: twice for 5 minutes at room temperature in 2× SSC, three times 10 minutes at 50°C in 2× SSC containing 0.4% SDS, 10 minutes at room temperature in 1× Washing Buffer (DIG Wash and Blocking Buffer Set, Roche). Membranes were blocked for 30 min at room temperature in 1× Blocking Buffer (DIG Wash and Blocking Buffer Set, Roche), then incubated with anti-Digoxigenin AP Fab fragments (Roche) diluted (1/10000) in 1× Blocking Buffer at room temperature for 30 min and then, they were washed twice (15 minutes each time) at room temperature in 1× Washing Buffer. Membranes were equilibrated in 1× detection buffer (DIG Wash and Blocking Buffer Set, Roche) incubate with several drops of CDP-star (Roche).

### RT-PCR

First, RNA was treated with DNase to eliminate any DNA contamination. In each sample, a total reaction of 10 μl contained: 500 ng RNA, 1μl *RQ1 RNase-Free DNase* (Promega), 1x *RQ1 DNase 10X Reaction Buffer* and DEPC water. Reactions were incubated for 30 min at 37°C. Reactions were stopped by adding 1 μl STOP solution (Promega) and incubating them for 10 min at 65°C. cDNA synthesis was performed using *Transcriptor First Strand cDNA Synthesis Kit* (Roche) from 500 ng of total RNA according to the manufacturer’s instructions. Samples were stored at 4°C for immediate use or at -20°C for longer periods. To study 3’ end processing, PCR amplification was performed using the *GoTaq DNA Polymerase* (Promega). The primers used were:

*sls-2*.*8* Fw 5’-GCTGTCGTTTCGATCTCTCG-3’;

Y75B8A.23 Rv 5’-TGTCGTGAGTAGGTGTGCAA-3’;

H27M09.8 Fw 5’-GTGTGGCAGTCTCGAGTTGA-3’;

H27MO9.5 Rv 5’-TTGAACCTTTTCGTCGGAAC-3’;

F08G2.9 Fw 5’-TGGAACCTAGGGAAGACTCG-3’;

*ins-37* Rv 5’-TTGAACTTGTCCGGGATTCT-3’;

W04G5.11 Fw 5’- ATTTTTGGAACCCAGGGAAG-3’;

W04G5.8 Rv 5’-GTGGAGATTTCTGCGACACA;

F08H9.10 Fw 5’- TGACCTATGTGGCAGTCTCG-3’;

F08H9.3 Rv 5’- TCGACAATCTCATTCCGACA-3’;

*act-1* Fw 5’- CCAGGAATTGCTGATCGTATG-3’;

*act-1* Rv 5’-GGAGAGGGAAGCGAGGATAG-3’

The final concentrations in each PCR reaction were: 1x *GoTaq Reaction Buffer* (1.5 mM MgCl_2_), 0.2 mM dNTPs, 0.2 μM upstream primer and downstream primer, 2.5 units *GoTaq DNA Polymerase* plus the required amount of DNA template (<500 ng) in each case. For the upstream primers that target the snRNAs, the concentration used was 0.8 μM.

Reactions were performed using GeneAmp PCR System 9700 thermal cyclers (Applied Biosystems). PCR conditions were adjusted in each reaction based on the DNA fragment to be amplified and the primer pairs used, but all reached 40 amplification cycles.

### cDNA Library Preparation and Ultrasequencing

Sequencing libraries were prepared by following the TruSeq RNA Library (LS) Preparation Kit v2 instructions (Illumina Inc., San Diego, CA) from 1 ug of total RNA that was previously depleted using the RiboZero (Human/Mouse/Rat) protocol. All libraries were run in a HiSeq1500 PE100 lane in Rapid mode, pooled in equimolar amounts to a 10 nM final concentration. The library concentration was measured via qPCR using the KAPA library quantification kit for Illumina sequencing platforms (Kapa Biosystems, Wilmington, MA) before high throughput sequencing.

**Bioinformatic analysis** was performed as described [48]: The quality of RNAseq results was initially assessed using FastQC (http://www.bioinformatics.babraham.ac.uk/projects/fastqc/). The raw reads were trimmed, filtered for those with a Phred quality score of at least 25 and all adapters were removed with Trimmomatic software [64].

Clean reads were aligned versus the N2 *Caenorhabditis elegans* reference genome (release WBcel235.85, http://www.ensembl.org/Caenorhabditis_elegans/Info/Index) using Tophat2 [65] with default parameters. Resulting alignment files were quality assessed with Qualimap2 [66] and sorted and indexed with Samtools software [67]. After taking a read count on gene features with the FeatureCounts tool [68], quantitative differential expression analysis between conditions was performed both by DESeq2 [69] and edgeR [70] implementations to compare the groups in pairs. Both implemented as R Bioconductor packages and performed read-count normalization by following a negative binomial distribution model. In order to automate this process and facilitate all group combination analysis, the SARTools pipeline [71] was used. All resultant data was obtained as HTML files and CSV tables, including density count distribution analysis, pairwise scatter plots, cluster dendrograms, Principal Component Analysis (PCoA) plots, size factor estimations, dispersion plots and MA- and volcano plots. Resulting tables, including raw counts, normalized counts, Fold-Change estimation and dispersion data for each of the analysis methods (DESeq2 and edgeR) were annotated with additional data from Biomart (https://bioconductor.org/packages/release/bioc/html/biomaRt.html), WormBase (http://www.wormbase.org) and org.Ce.eg.db (https://bioconductor.org/packages/release/data/annotation/html/org.Ce.eg.db.html) databases. Final tables also include the associated gene name, Ensembl Transcript and protein information, GO Term ID and names, EntrezID, UniprotTrEMBL information and Human ortholog ID and gene name data.

In order to control the False Discovery Rate (FDR), p-values were amended by Benjamini-Hochberg (BH) multiple testing corrections [72]. Those features showing corrected p-values below the 0.05 threshold were considered up- or down-regulated genes. To reinforce downstream analysis and discard false-positive over/under-expressed genes, common up- and down-regulated features were extracted from DESeq2 and edgeR tables. The CRAN packages eVenn (https://www.rdocumentation.org/packages/eVenn/versions/2.4) and pheatmap (https://CRAN.R-project.org/package=pheatmap) were used to graphically represent Venn diagrams and heatmap plots showing these common features.

Gene Ontology enrichment analysis was performed for common up/down regulated genes by using the clusterProfiler package [73] through its enrichGO tool. This tool uses a hypergeometric BH model to obtain adjusted q-values. Each GO category (Biological Process–BP-, Molecular Function–MF, and Cellular Component–CC-) was represented in bar plots, showing its relative abundance and associated q-value. Similarly, KEGG and Reactome pathway analysis was conducted using clusterProfiler (enrichKEGG) and ReactomePA [74] tools. The KEGG pathway maps were obtained with the Pathview package [75].

By tacking DESeq2 expression values, the regularized log2 transformation (rlog) data was represented as a 2-dimensional Principal Component Analysis (PCA) plot. Sample-to-sample Euclidean distances were calculated from the rld data and represented in a heatmap, showing the adjacent clustering information [48].

Lists of up- and downregulated genes are available online for comparison and easy visualization by loading the following datasets onto the web version of the Upset application (http://caleydo.org/tools/upset/):

Upregulated genes:

https://raw.githubusercontent.com/CharoLopez/upset-data/master/Up_regulated_all.json

Downregulated genes:

https://raw.githubusercontent.com/CharoLopez/upset-data/master/Down_regulated_all.json

## Supporting information

S1 Fig Fertility and viability of N2 vs. the *t1903* mutant. (**a**) Fertility of N2 vs. the *t1903* mutant at 15°C and 25°C. The graph shows the number of eggs laid by N2 worms (in black; n = 10) compared to those laid by *t1903* worms (in gray; n = 10) growing at 15°C and 25°C (Mean ±standard error of the mean (sem)). The differences between N2 and *t1903* are statistically significant. P-values correspond to the Student’s t-test. (**b**) Embryonic viability (%) of N2 versus *t1903* mutants, growing at 15°C and 25°C. The graph shows the percentage of hatched larvae in N2 worms (black) compared to the percentage of hatched larvae in *t1903* worms (gray) growing at 15°C and 25°C.
(TIF)

S2 Fig Western blot validation of the INTS-6::3xFLAG::GFP full length protein formation. (**a**) Upper panel shows anti-GFP detection/quantification of the tagged INTS-6 protein under control of both the *eft-3* promoter and the endogenous *ints-6* promoter. Size corresponds to that expected for INTS-6 (98kDa) plus 3xFLAG (2.6kDa) and GFP (28kDa). Lower panel shows actin as loading control. (**b**) (**c**) Entire western blot shows INTS-6::3xFLAG::GFP as the only protein detected, with no significant degradation or cleavage fragments found using anti-GFP and anti-FLAG antibodies.
(TIF)

S3 Fig Human INTS6 localization. (**a**) Schematic representation of the plasmids, pBS15 and pBS16, used for transfection. (**b**) INTS6 *in vivo* localization in 293T cells transfected with pBS15 or pBS16 (**c**) INTS6 *in vivo* localization in U2OS cells transfected with pBS15 or pBS16.
(TIF)

S4 Fig *C. elegans* INTS-6 localization. Immunostaining of: JCP383 *ints-6* (*tm1615*) IV; jcpSi10[pJC51(*ints-6*p::*ints-6*::3xFLAG::eGFP::*ints-6*UTR,*unc-119*(+))] II using antiFLAG antibodies (left panel) and DAPI (right panel). *C*. *elegans* INTS-6 shows a mainly nuclear localization in early embryos (1 and 2), middle-late embryos (3), and adults (head (4), gonad (5), gut (6) and tail (7)).
(TIF)

S5 Fig polyA RNA seq experiments show that *ints-6* (*t1903*) has snRNA processing defects. snRNAs are non-polyadenylated and therefore practically not detected in the polyA RNAseq analysis of WT N2 control (upper panel). Reads corresponding to the snRNA, the downstream intergenic region and the downstream gene are detected in the *ints-6* (*t1903*) mutant at 15°C and, at a higher level, at the restrictive temperature, 25°C. Reads corresponding to the chimeric sn-mRNA are polyadenylated and therefore detected in the polyA assay. As expected for polyA RNAseq, the 3’ end of the gene is enriched.
(TIF)

S6 Fig *ints-6* (*t1903*) mutant disrupts processing of SL snRNAs. RNA deep sequencing reads aligned to the *C*. *elegans* genome in the regions of SL snRNA genes, visualized on IGV software. N2 reads are shown in gray whereas *ints-6* (*t1903)* mutant reads are in black. Underneath each graph, the *C*. *elegans* genome is represented in blue. The exons are shown as blue boxes and the introns as lines. (**a**) Shows a region of the *C*. *elegans* chromosome V where *sls-1* genes cluster paired with rRNAs genes. (**b**) Shows the *C*. *elegans* chromosome II in the region of the gene *sls-2*.*1*. In the WT, SL snRNAs are processed and trans-spliced as short exons to coding mRNAs. Therefore, their reads do not fully match to the SL loci and the alignment is low. In contrast, unprocessed SL snRNAs reads align to their coding and downstream region and therefore are fully detected.
(TIF)

S7 Fig Full list of INTS-6-associated proteins identified by anti-FLAG affinity purification. In addition to members of the Integrator complex, other proteins were immunoprecipitated along with INTS-6::3xFLAG::GFP. Proteins found in the FLAG affinity eluate that also appeared in the control using WT N2 animals were discarded as nonspecific binding. RNAPII was not immunoprecipitated with INTS-6, suggesting an indirect interaction in the complex. In humans, the interaction between both complexes is mediated by INTS1 and the C terminal domain of the RNAPII.
(TIF)

S8 Fig Validation of RNAi depletion of Integrator subunits. (**a**) RNA-dependent RNA polymerase (RDRP) mediated amplification of the different integrator members, shown as a red line, indicates efficient dsRNA interference. (**b**) RNAi Integrator subunits depletion was efficient in the 3 different transcriptional classes. Western blots of representative subunits of the Auxiliary, Holder and Catalytic classes tagged with 3xFLAG show a strong depletion of the proteins upon RNAi treatment. In all cases, the upper panel shows the tagged protein in the control L4440 RNAi vs specific RNAi, visualized with anti-FLAG. The lower panels show actin as the loading control.
(TIF)

S9 Fig **Northern blot analysis for U1 snRNA (a) and U2 snRNA (b) from *C*. *elegans* worms six days after treatment with RNAi L4440 (control), RNAi of *ints-6*, N2 and *ints-6 (t1903)* mutant grown o/n at 25**°**C**. Mature snRNA is detected after six days of *ints-6* silencing. U6 snRNA is shown as a control. Knockdown of *ints-6* and the *t1903* mutation lead to generation of chimeric sn-mRNAs (**c**, **d**). Probes from either internal region of U1 snRNA and U2 snRNA or the 3’ region of snRNA are shown for each blot.
Capture of the corresponding RNA-seq alignment reads shows the contribution of chimeric sn-mRNA (in *ints-6 t1903* mutant or RNAi) versus the normal expression of gene mRNA (in an empty L4440 RNAi vector or a WT N2) (**e,f**).
(TIF)

S10 Fig Quantification of the snRNA 3’ end processing defects upon knockdown of the Integrator subunits. Expression levels of U1 (**a**) or U2 (**b**) snRNAs are not significantly affected by RNAi of the different integrator subunits. Normalized counts for snRNA gene expression of the 3 replicas show no statistical differences between the control and the different RNAi integrator subunits. U1 and U2 are properly processed at their 3’ ends in the control (**c** and **d**). RNAi Knockdown of *C*. *elegans* Integrator subunits leads to no more than a 1.4% lack of U1 3’ end processing (**c**) and up to a 6.2% lack of U2 3’ end processing.
(TIF)

S11 Fig Read-through transcription downstream of the snRNA loci reaches the expression level of regulatory genes such as *lit-1*/NLK or *daf-16*/FOXO of the wnt and insulin pathways respectively, after knockdown the Integrator complex. Normalized expression data of the *act-1*/actin, atm-1/ATM *ced-1*/MEGF11, *daf-16*/FOXO, *egl-1*/BH3 and *lit-1*/NLK genes are shown for the control and the knockdown of different Integrator subunits. U1 and U2 read-through are absent in the control but reach a physiological level after Integrator subunit RNAi.
(TIF)

S12 Fig Quantification of splicing defects caused by RNAi knockdown of the Integrator subunits in *C. elegans*. Intron retention was determined as the ratio between reads in the gene introns versus total reads of the gene (mean ± standard error of the mean). (**a**) Shows intron retention of genes located directly downstream of snRNA loci, and therefore transcribed as chimeric sn-mRNAs. Analyzed genes: F08H9.3, C15F1.5, H27M09.5, F58G1.7, F08H9.12, T08G5.3, W04G5.8, F15H9.3, F15H9.4, R05D7.3, F56H6.2, Y54G9A.4, F08G2.6, F08G2.8, Y57G11C.5. Expression of these genes in the WT was low and no intron retention was detected. As a control, (**b**) shows intron retention of genes located upstream of the snRNA loci, and therefore not transcribed as chimeric sn-mRNAs. Analyzed genes: F08H9.4, C15F1.6, H27M09.3, Y38F1A.1, F08H9.6, T08G5.5, W04G5.2, M01G12.9, R05D7.1, R05D7.4, F41D3.5, Y54G9A.5, F08G2.7, Y57G11.4. Significant differences in a T-student test are shown with asterisks.
(TIF)

S13 Fig Detection of antisense RNAs derived from the lack of processing of snRNAs located *in antisense* downstream of coding genes. Directional RNAseq alignments of WT and *ints-6* (*t1903*) mutant worms to the *C*. *elegans* reference genome. Reads on the + strand are shown in blue and reads on the–strand are shown in red. The black line marks the 3’ end of the snRNA.
For each case, the upper track shows the genomic region of snRNA loci located downstream and opposite to coding genes. The middle track shows the RNAseq alignment of WT worms. RNAseq shows only the mRNA and the mature snRNA. The lower track shows the RNAseq alignment of *ints-6* (*t1903*) mutant worms. Both types of transcripts are present: mRNA and *antisense* RNAs on the opposite strand, derived from the lack of processing of snRNAs located *in antisense* downstream of the gene. (**a**) Shows the gene *glc-4*, (**b**) the gene W06D4.2, (**c**) the gene rpt-1 and (**d**) the gene W09D6.5.
(TIF)

S14 Fig “Chimeric U1 and U2-mRNAs” are not translated into peptides. (**a**) Scheme of the plasmids made and integrated into chromosome II, using the mosSCI system. Plasmid pJC63 was used to generate the JCP479 transgenic strain. A genomic region amplified upstream of U1 F08H9.10 to the downstream region of the F08H9.3 gene is shown. A cassette with the HA, MYC (+1b) and TY (+2b) tags in each of the 3 ORFs was inserted into U1 F08H9.3 so that the HA tag and the MYC tag were in-frame respectively with the first and the second ATGs of the U1 F08H9.10. Plasmid pJC64 was used to generate the JCP504 strain. A genomic region amplified upstream of U2 W04G5.11 to the downstream region of the W04G5.8 gene is shown. The same cassette with the HA, MYC (+1b) and TY (+2b) tags in each of the 3 ORFs was inserted into the U2 W04G5.11. (**b**) WBs of protein extracts from the JCP479 and JCP504 transgenic strains after RNAi treatment of the *C*. *elegans* Integrator complex subunits *ints-2*, *-9* and *-11* and the empty L4440 vector. Expected molecular weights: JCP479/JCP504 for HA (1^st^ ORF): 7 kDa; MYC (2^nd^ ORF): 8.7 kDa; TY (3^rd^ ORF): 5.5 kDa. Positive control HA: 28.4 kDa; positive control MYC: 61.5 kDa; positive control TY: 54.3 kDa.
(TIF)

S15 Fig Sample-to-sample distance heatmap between worm samples. Sample-to-sample distance heatmap showing the Euclidean distances (calculated from the rld data) between worm samples. Upper and left-side dendrograms show samples grouped by similarity of their transcriptional profiles.
(TIF)

S1 Table Main ncRNA types in *C. elegans*. **Analysis of the influence on the 3’-end processing when any of the Integrator complex subunits are knocked down.** For each ncRNA type, the following information is shown: gene name, chromosome (Chr), genomic position, U Strand (sense: 1; antisense: -1), 3’-end processing affected (yes/no), name of downstream gene affected, strand of downstream gene affected (sense: 1; antisense: -1) and snRNA localization inside of an intron (yes/no).
(XLS)

S2 Table Integrator subunits in *H. sapiens* and their orthologs in the following species: *M. musculus*, *G. gallus*, *D. rerio*, *D. melanogaster* and *C. elegans*. The Integrator subunit protein sequences were searched for in the Uniprot database (http://www.uniprot.org/) and their corresponding orthologs were searched for using the BLASTp tool with default parameters against the corresponding genome (https://blast.ncbi.nlm.nih.gov/Blast.cgi). Results were corroborated at www.orthodb.org. Percentages of identity (in bold) and similarity (in brackets) are shown. Each cell in the table is colored depending on the percentage of identity between the human sequence and the organism being considered. Black means 100% homology and white means no significant homology. Asterisks indicate that significant homology was found on a portion covering less than 50% of the protein sequence.
(XLSX)

S3 Table snRNA sequences. Sequence of the different snRNAs in *C*. *elegans*. CLUSTAL multiple sequence alignment by MUSCLE (3.8) and consensus sequence of each snRNA type.
(DOC)

S4 Table Multipage excel file showing the lists of genes significantly up- or downregulated in each group. (XLS)

S5 Table Gene Ontology (GO) enrichment analysis of genes specifically affected after RNAi knockdown of each Integrator complex member. The cellular component and molecular function of the corresponding up-regulated and down-regulated genes are shown.
(XLSX)
