# Supplementary figures and images for: Disruption of the Caenorhabditis elegans Integrator complex triggers a non-conventional transcriptional mechanism beyond snRNA genes
Source: PLoS Genet. 2019 Feb 26;15(2):e1007981. doi: 10.1371/journal.pgen.1007981 (PMC6390993; doi:10.1371/journal.pgen.1007981)

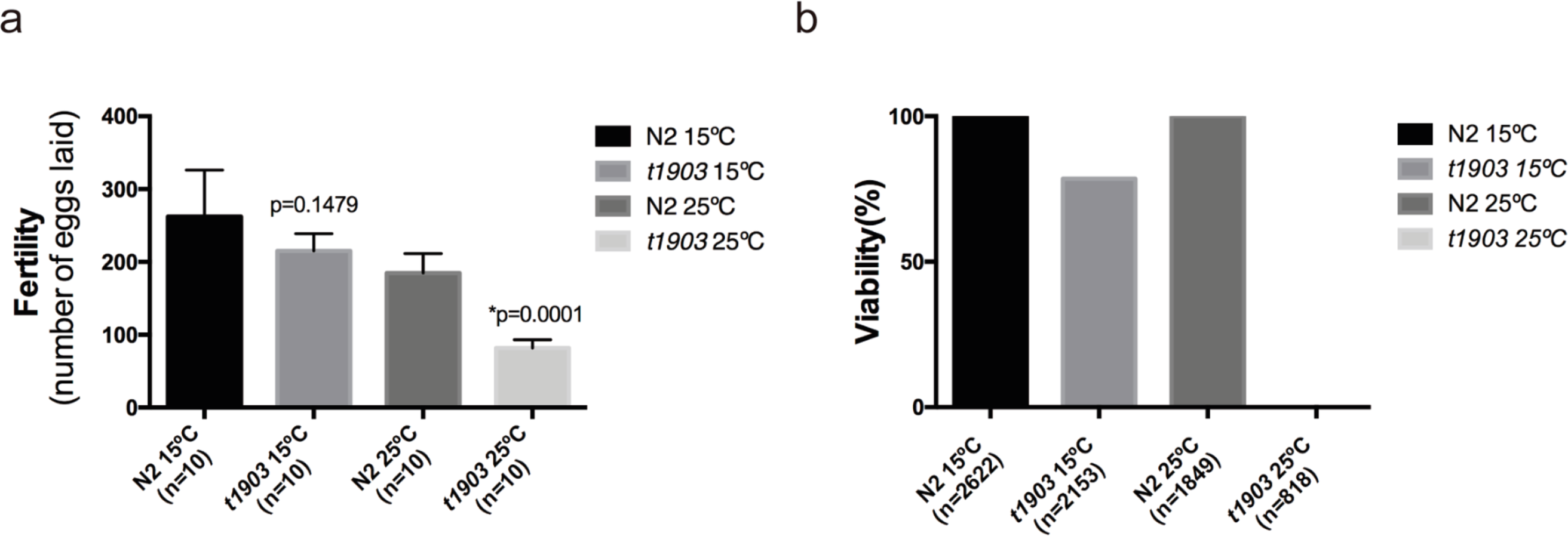

Supplement: S1 Fig — (a) Fertility of N2 vs. the t1903 mutant at 15°C and 25°C. The graph shows the number of eggs laid by N2 worms (in black; n = 10) compared to those laid by t1903 worms (in gray; n = 10) growing at 15°C and 25°C (Mean ±standard error of the mean (sem)). The differences between N2 and t1903 are statistically significant. P-values correspond to the Student’s t-test. (b) Embryonic viability (%) of N2 versus t1903 mutants, growing at 15°C and 25°C. The graph shows the percentage of hatched larvae in N2 worms (black) compared to the percentage of hatched larvae in t1903 worms (gray) growing at 15°C and 25°C. (TIF) [file pgen.1007981.s001.tif]

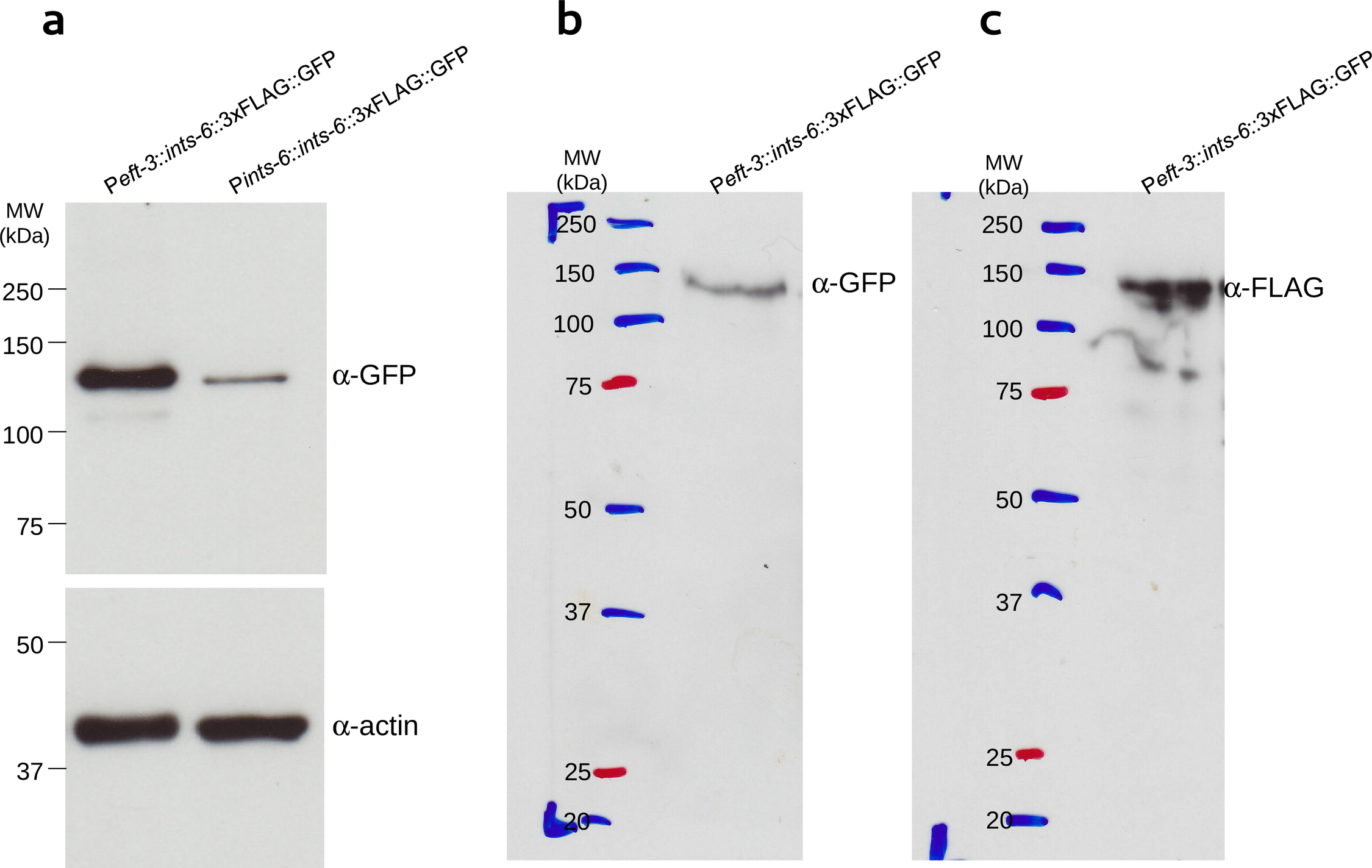

Supplement: S2 Fig — (a) Upper panel shows anti-GFP detection/quantification of the tagged INTS-6 protein under control of both the eft-3 promoter and the endogenous ints-6 promoter. Size corresponds to that expected for INTS-6 (98kDa) plus 3xFLAG (2.6kDa) and GFP (28kDa). Lower panel shows actin as loading control. (b) (c) Entire western blot shows INTS-6::3xFLAG::GFP as the only protein detected, with no significant degradation or cleavage fragments found using anti-GFP and anti-FLAG antibodies. (TIF) [file pgen.1007981.s002.tif]

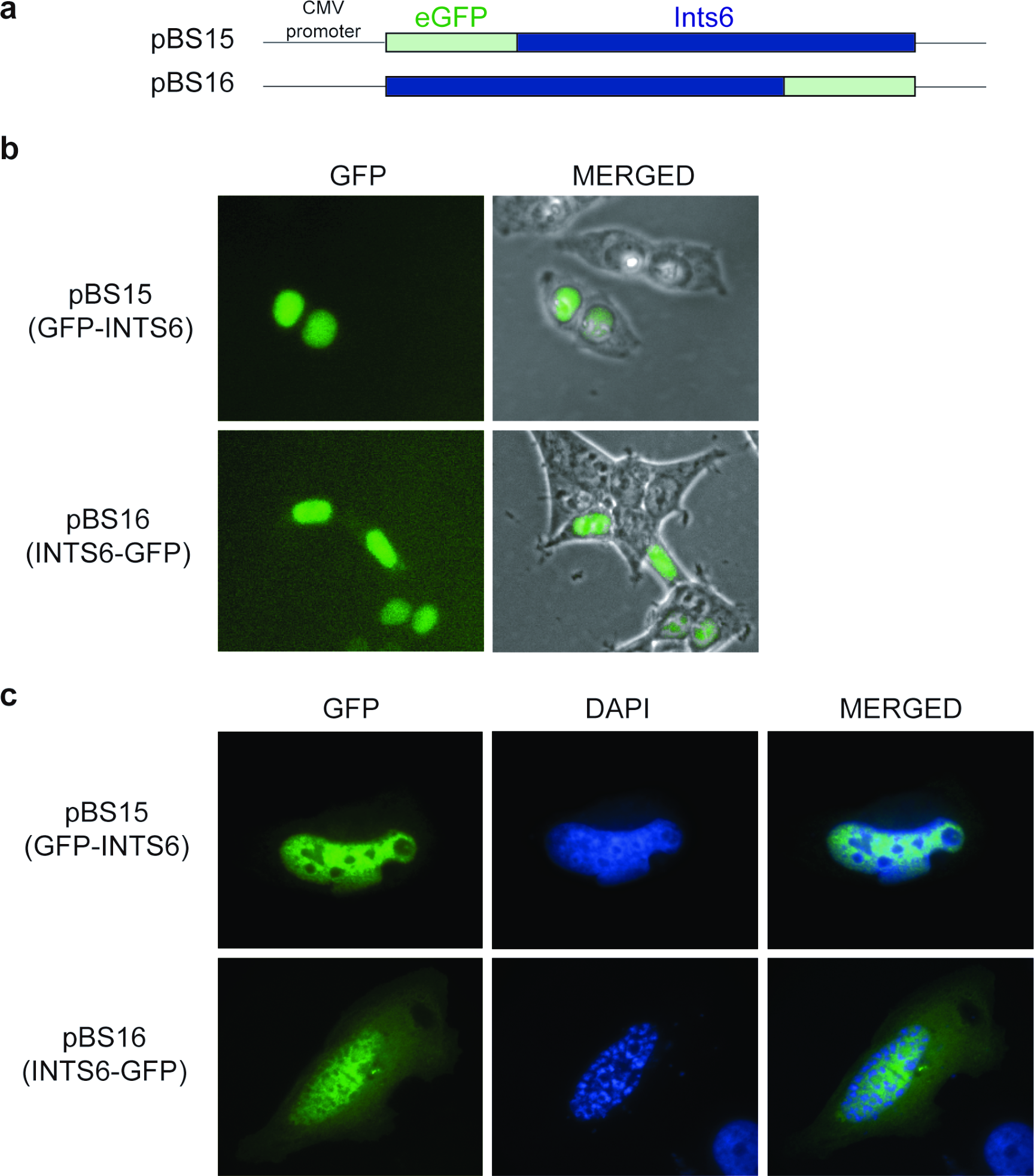

Supplement: S3 Fig — (a) Schematic representation of the plasmids, pBS15 and pBS16, used for transfection. (b) INTS6 in vivo localization in 293T cells transfected with pBS15 or pBS16 (c) INTS6 in vivo localization in U2OS cells transfected with pBS15 or pBS16. (TIF) [file pgen.1007981.s003.tif]

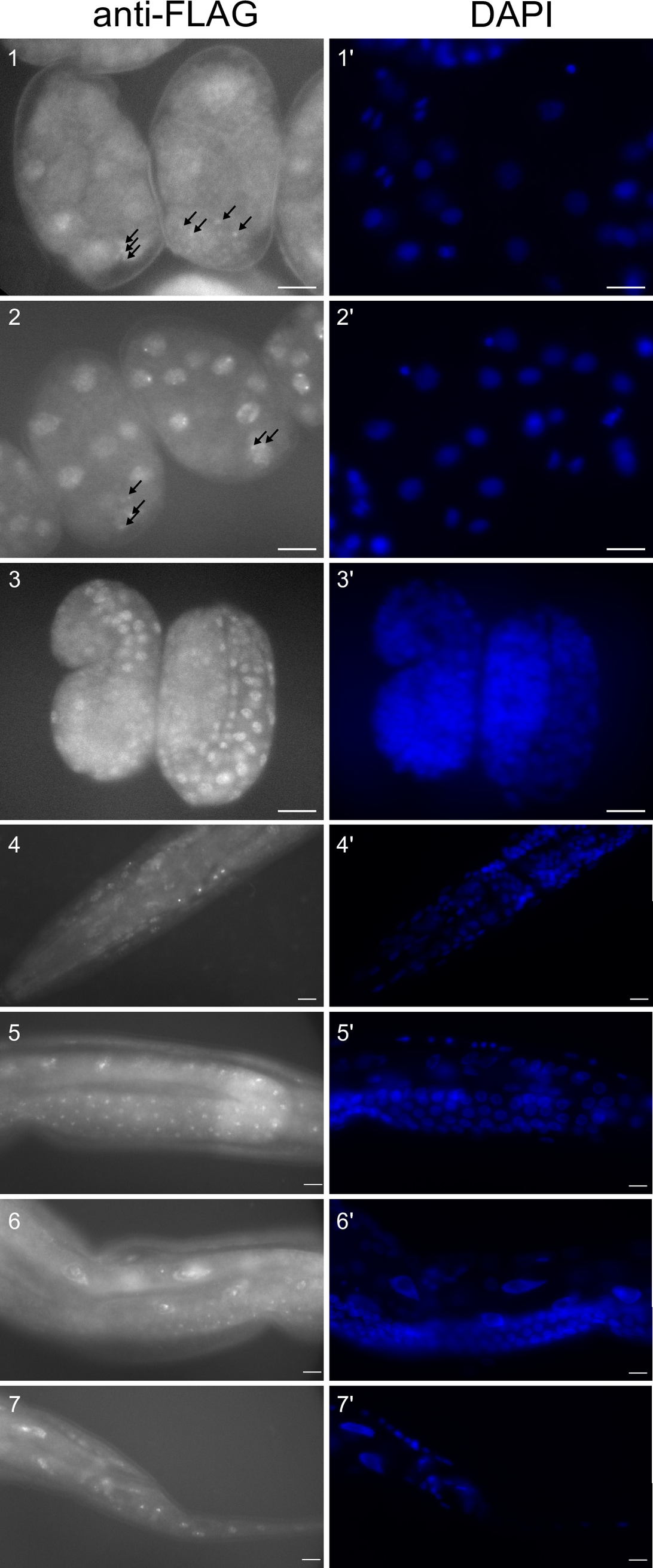

Supplement: S4 Fig — Immunostaining of: JCP383 ints-6 (tm1615) IV; jcpSi10[pJC51(ints-6p::ints-6::3xFLAG::eGFP::ints-6UTR,unc-119(+))] II using antiFLAG antibodies (left panel) and DAPI (right panel). C. elegans INTS-6 shows a mainly nuclear localization in early embryos (1 and 2), middle-late embryos (3), and adults (head (4), gonad (5), gut (6) and tail (7)). (TIF) [file pgen.1007981.s004.tif]

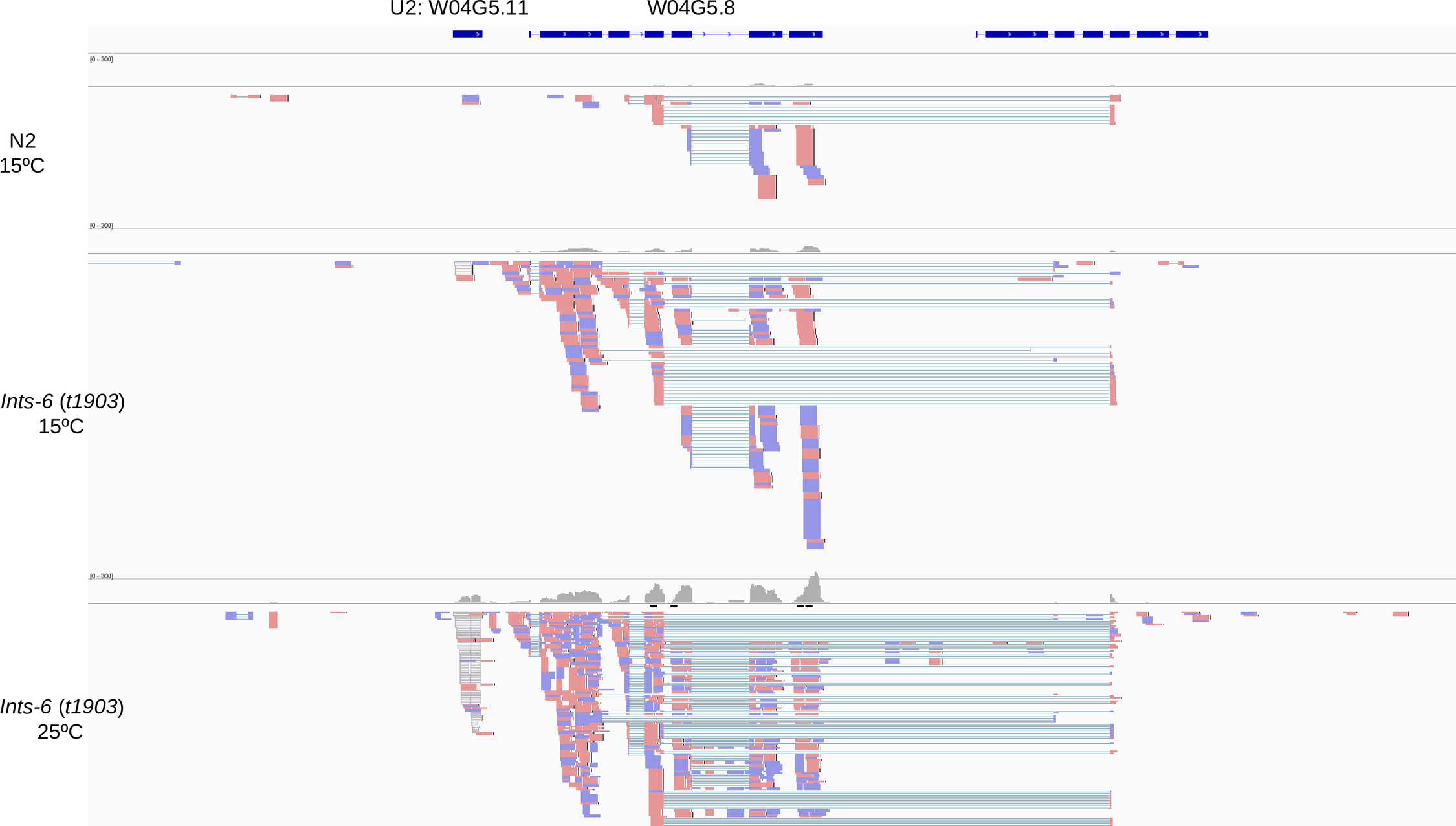

Supplement: S5 Fig — snRNAs are non-polyadenylated and therefore practically not detected in the polyA RNAseq analysis of WT N2 control (upper panel). Reads corresponding to the snRNA, the downstream intergenic region and the downstream gene are detected in the ints-6 (t1903) mutant at 15°C and, at a higher level, at the restrictive temperature, 25°C. Reads corresponding to the chimeric sn-mRNA are polyadenylated and therefore detected in the polyA assay. As expected for polyA RNAseq, the 3’ end of the gene is enriched. (TIF) [file pgen.1007981.s005.tif]

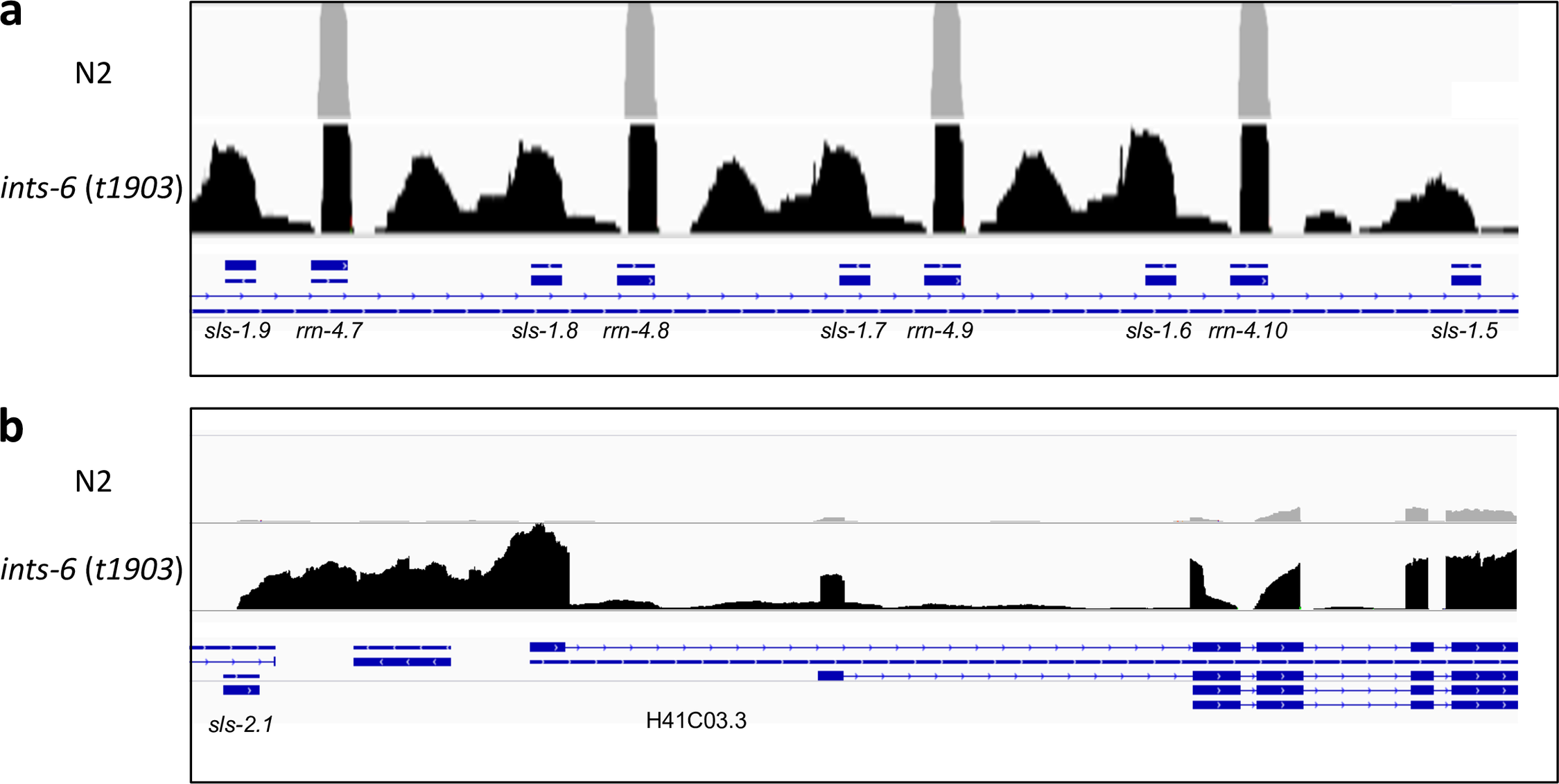

Supplement: S6 Fig — RNA deep sequencing reads aligned to the C. elegans genome in the regions of SL snRNA genes, visualized on IGV software. N2 reads are shown in gray whereas ints-6 (t1903) mutant reads are in black. Underneath each graph, the C. elegans genome is represented in blue. The exons are shown as blue boxes and the introns as lines. (a) Shows a region of the C. elegans chromosome V where sls-1 genes cluster paired with rRNAs genes. (b) Shows the C. elegans chromosome II in the region of the gene sls-2.1. In the WT, SL snRNAs are processed and trans-spliced as short exons to coding mRNAs. Therefore, their reads do not fully match to the SL loci and the alignment is low. In contrast, unprocessed SL snRNAs reads align to their coding and downstream region and therefore are fully detected. (TIF) [file pgen.1007981.s006.tif]

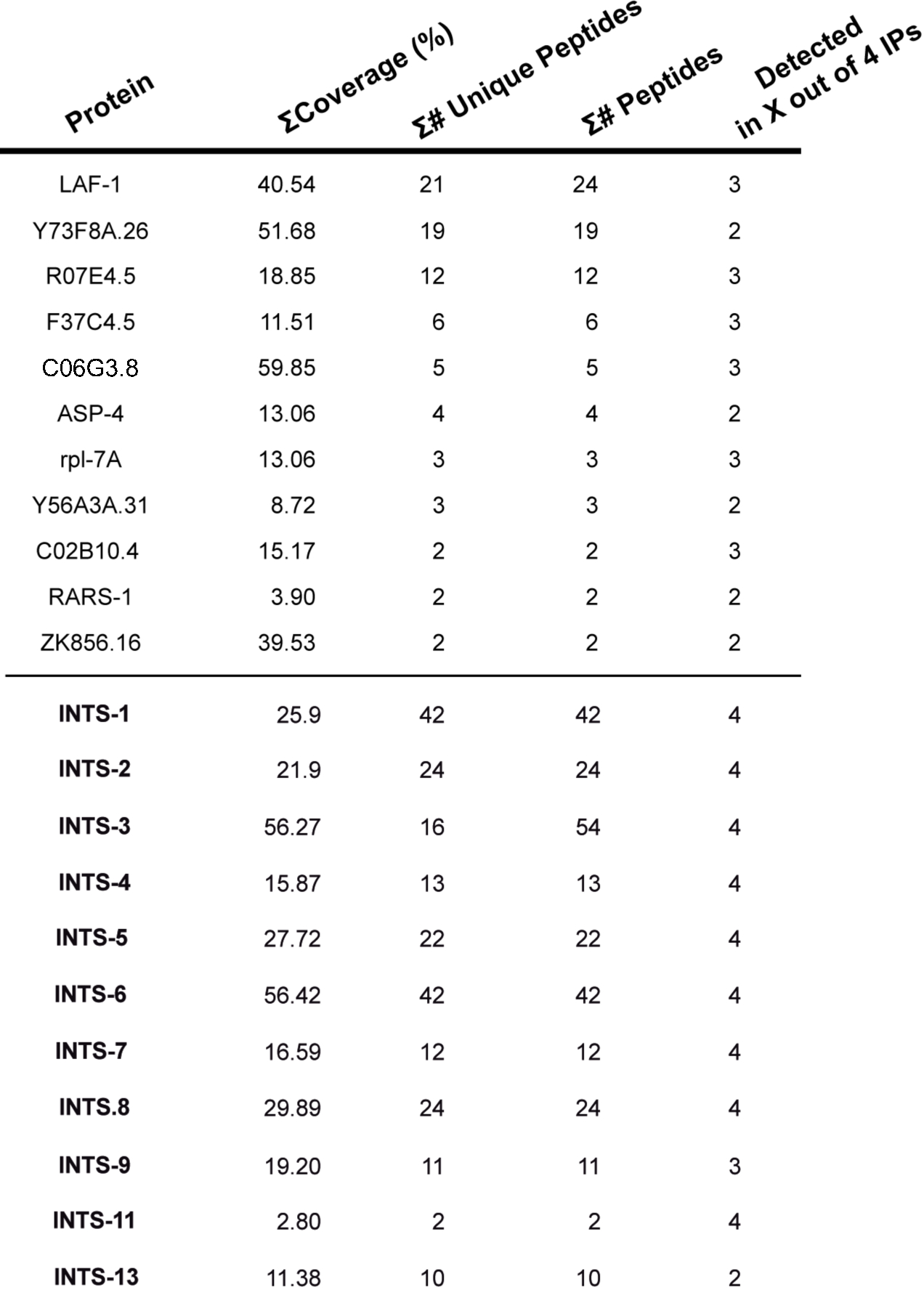

Supplement: S7 Fig — In addition to members of the Integrator complex, other proteins were immunoprecipitated along with INTS-6::3xFLAG::GFP. Proteins found in the FLAG affinity eluate that also appeared in the control using WT N2 animals were discarded as nonspecific binding. RNAPII was not immunoprecipitated with INTS-6, suggesting an indirect interaction in the complex. In humans, the interaction between both complexes is mediated by INTS1 and the C terminal domain of the RNAPII. (TIF) [file pgen.1007981.s007.tif]

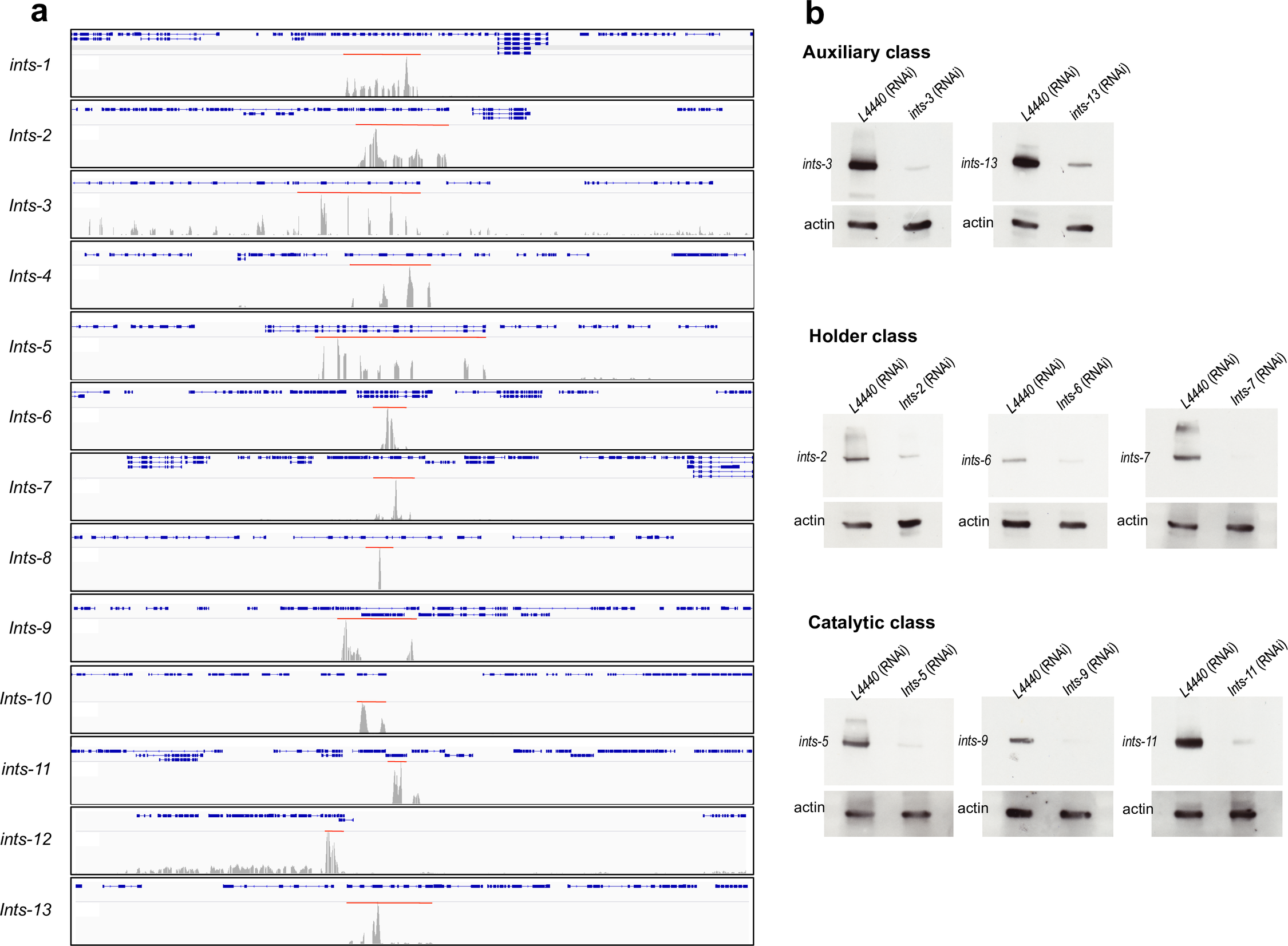

Supplement: S8 Fig — (a) RNA-dependent RNA polymerase (RDRP) mediated amplification of the different integrator members, shown as a red line, indicates efficient dsRNA interference. (b) RNAi Integrator subunits depletion was efficient in the 3 different transcriptional classes. Western blots of representative subunits of the Auxiliary, Holder and Catalytic classes tagged with 3xFLAG show a strong depletion of the proteins upon RNAi treatment. In all cases, the upper panel shows the tagged protein in the control L4440 RNAi vs specific RNAi, visualized with anti-FLAG. The lower panels show actin as the loading control. (TIF) [file pgen.1007981.s008.tif]

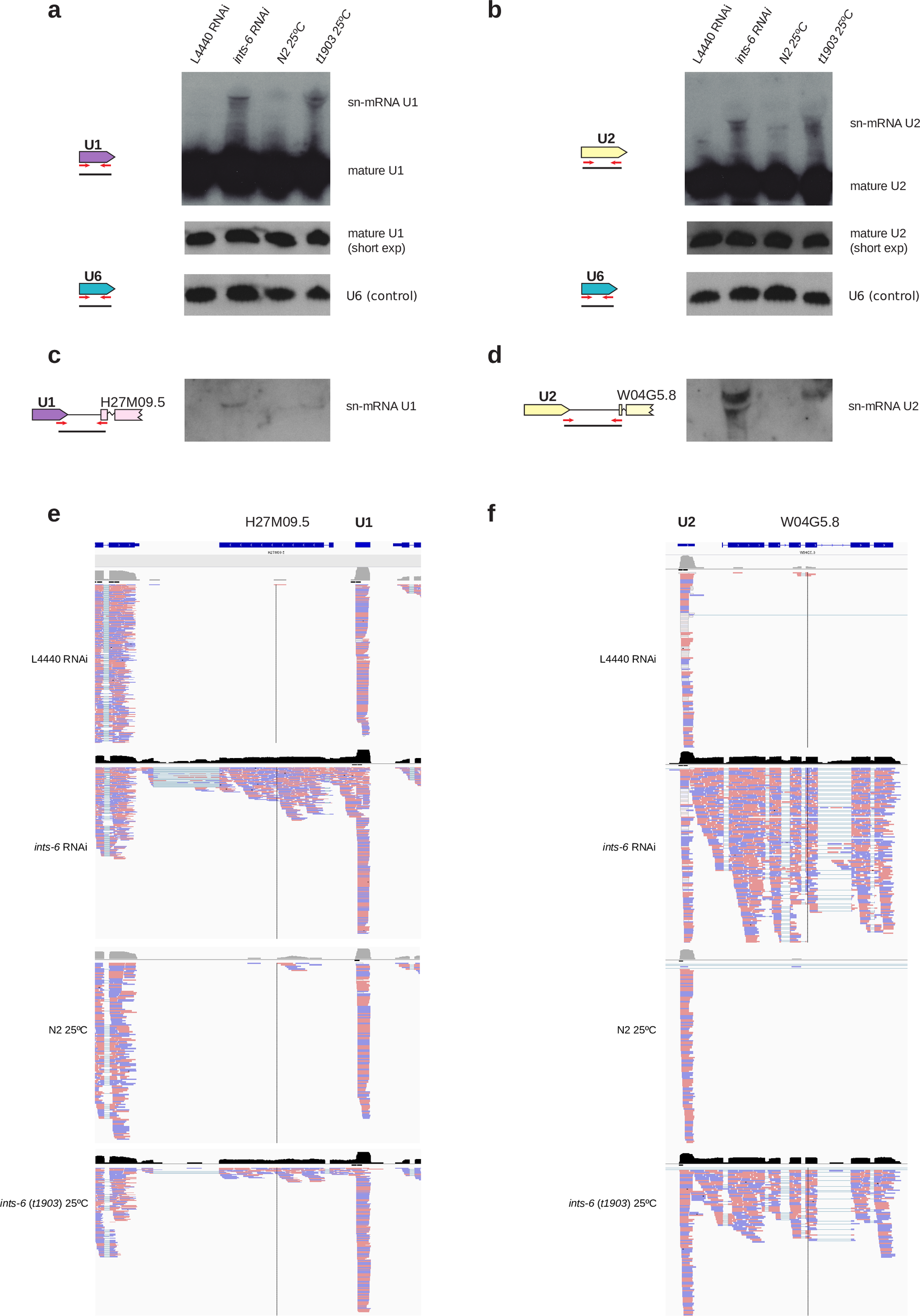

Supplement: S9 Fig — Northern blot analysis for U1 snRNA (a) and U2 snRNA (b) from C. elegans worms six days after treatment with RNAi L4440 (control), RNAi of ints-6, N2 and ints-6 (t1903) mutant grown o/n at 25°C. Mature snRNA is detected after six days of ints-6 silencing. U6 snRNA is shown as a control. Knockdown of ints-6 and the t1903 mutation lead to generation of chimeric sn-mRNAs (c, d). Probes from either internal region of U1 snRNA and U2 snRNA or the 3’ region of snRNA are shown for each blot. Capture of the corresponding RNA-seq alignment reads shows the contribution of chimeric sn-mRNA (in ints-6 t1903 mutant or RNAi) versus the normal expression of gene mRNA (in an empty L4440 RNAi vector or a WT N2) (e,f). (TIF) [file pgen.1007981.s009.tif]

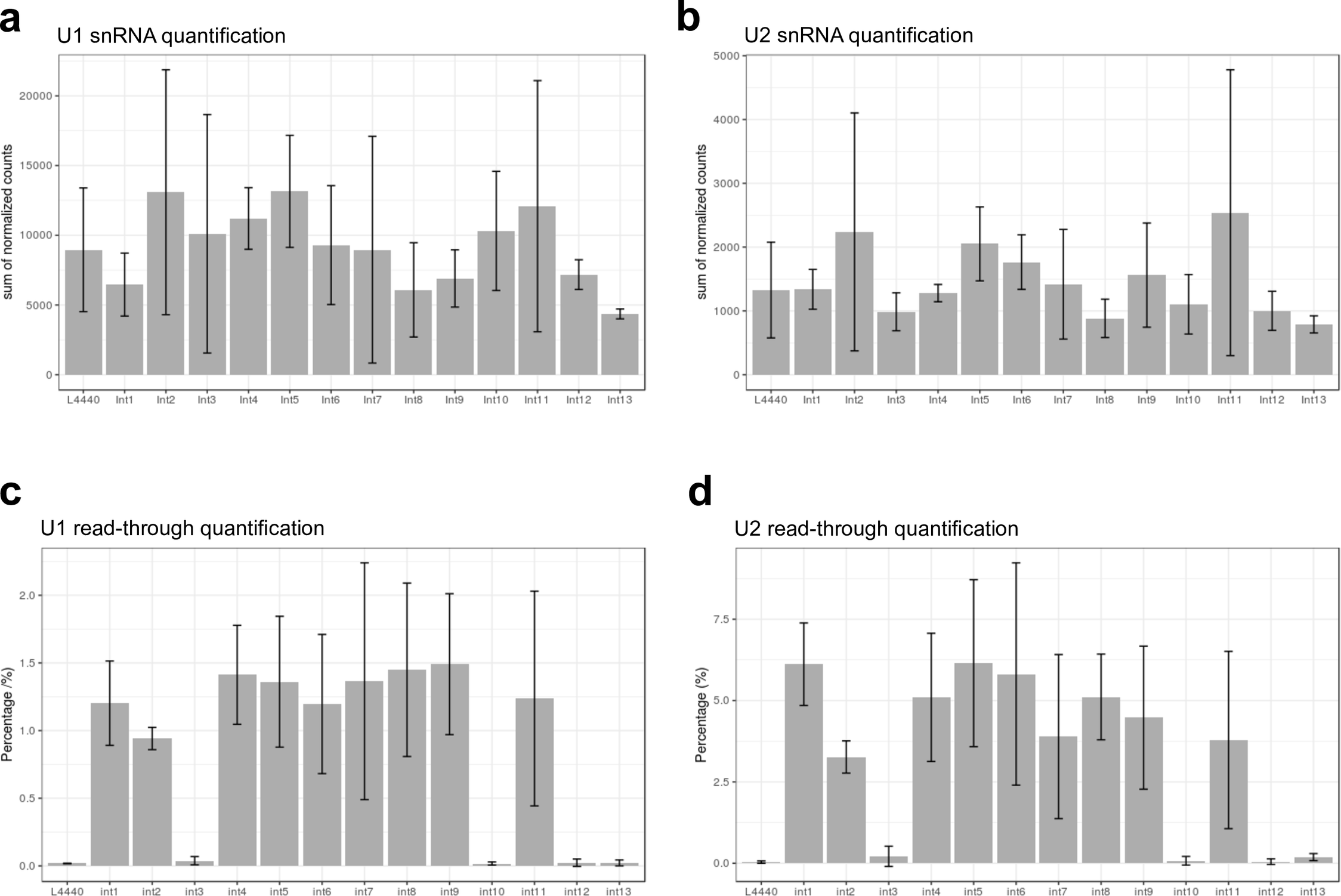

Supplement: S10 Fig — Expression levels of U1 (a) or U2 (b) snRNAs are not significantly affected by RNAi of the different integrator subunits. Normalized counts for snRNA gene expression of the 3 replicas show no statistical differences between the control and the different RNAi integrator subunits. U1 and U2 are properly processed at their 3’ ends in the control (c and d). RNAi Knockdown of C. elegans Integrator subunits leads to no more than a 1.4% lack of U1 3’ end processing (c) and up to a 6.2% lack of U2 3’ end processing. (TIF) [file pgen.1007981.s010.tif]

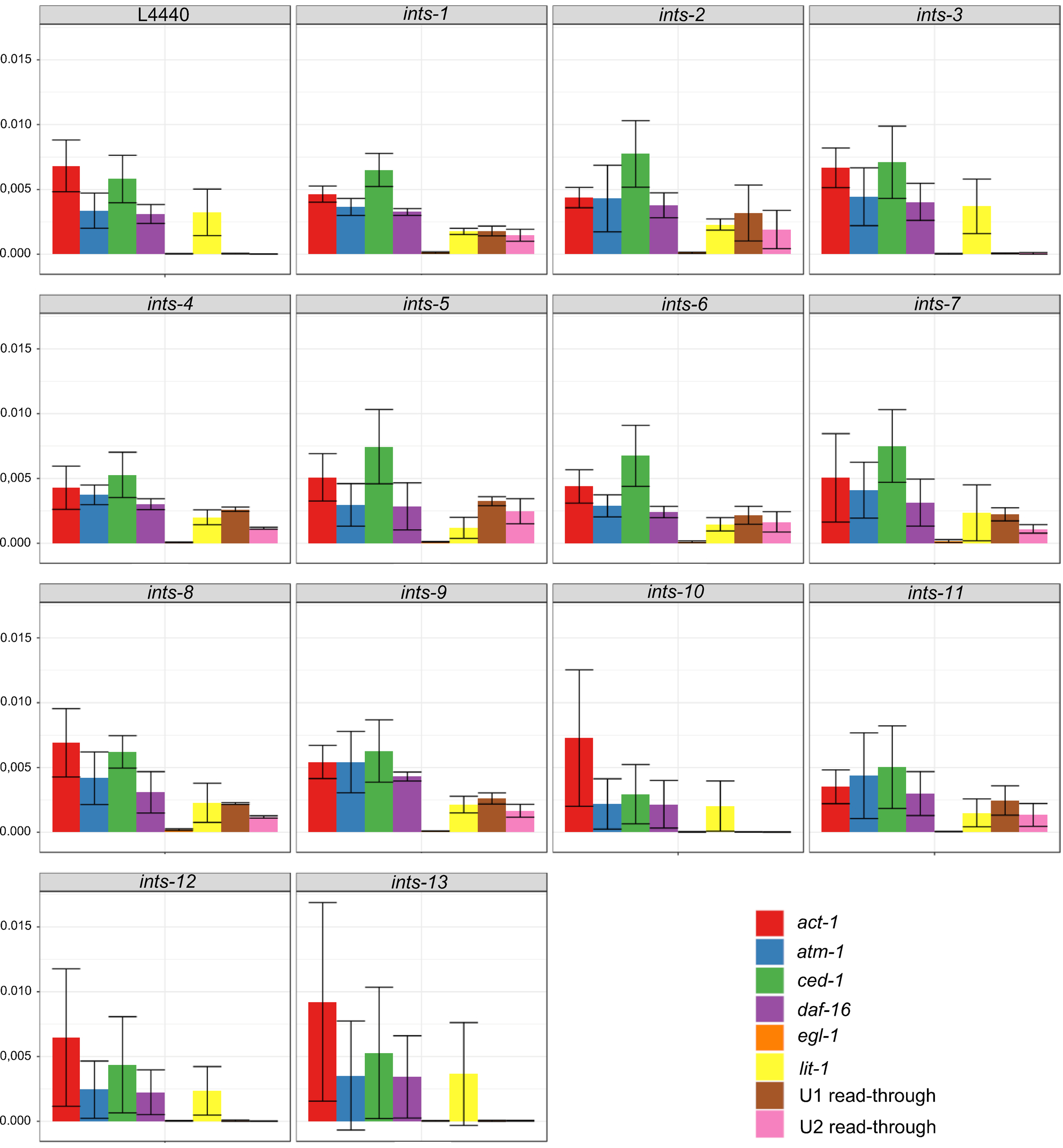

Supplement: S11 Fig — Normalized expression data of the act-1/actin, atm-1/ATM ced-1/MEGF11, daf-16/FOXO, egl-1/BH3 and lit-1/NLK genes are shown for the control and the knockdown of different Integrator subunits. U1 and U2 read-through are absent in the control but reach a physiological level after Integrator subunit RNAi. (TIF) [file pgen.1007981.s011.tif]

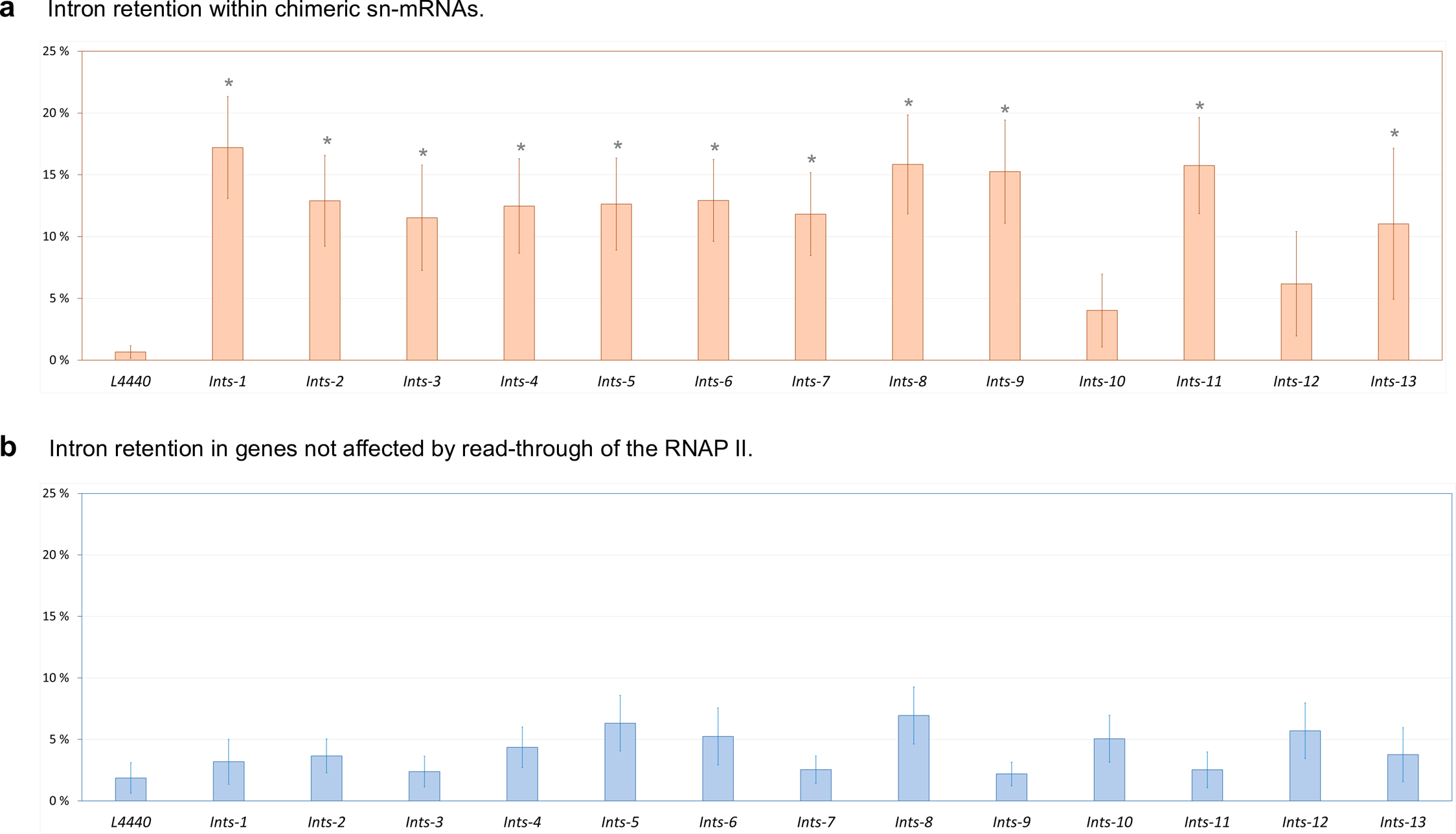

Supplement: S12 Fig — Intron retention was determined as the ratio between reads in the gene introns versus total reads of the gene (mean ± standard error of the mean). (a) Shows intron retention of genes located directly downstream of snRNA loci, and therefore transcribed as chimeric sn-mRNAs. Analyzed genes: F08H9.3, C15F1.5, H27M09.5, F58G1.7, F08H9.12, T08G5.3, W04G5.8, F15H9.3, F15H9.4, R05D7.3, F56H6.2, Y54G9A.4, F08G2.6, F08G2.8, Y57G11C.5. Expression of these genes in the WT was low and no intron retention was detected. As a control, (b) shows intron retention of genes located upstream of the snRNA loci, and therefore not transcribed as chimeric sn-mRNAs. Analyzed genes: F08H9.4, C15F1.6, H27M09.3, Y38F1A.1, F08H9.6, T08G5.5, W04G5.2, M01G12.9, R05D7.1, R05D7.4, F41D3.5, Y54G9A.5, F08G2.7, Y57G11.4. Significant differences in a T-student test are shown with asterisks. (TIF) [file pgen.1007981.s012.tif]

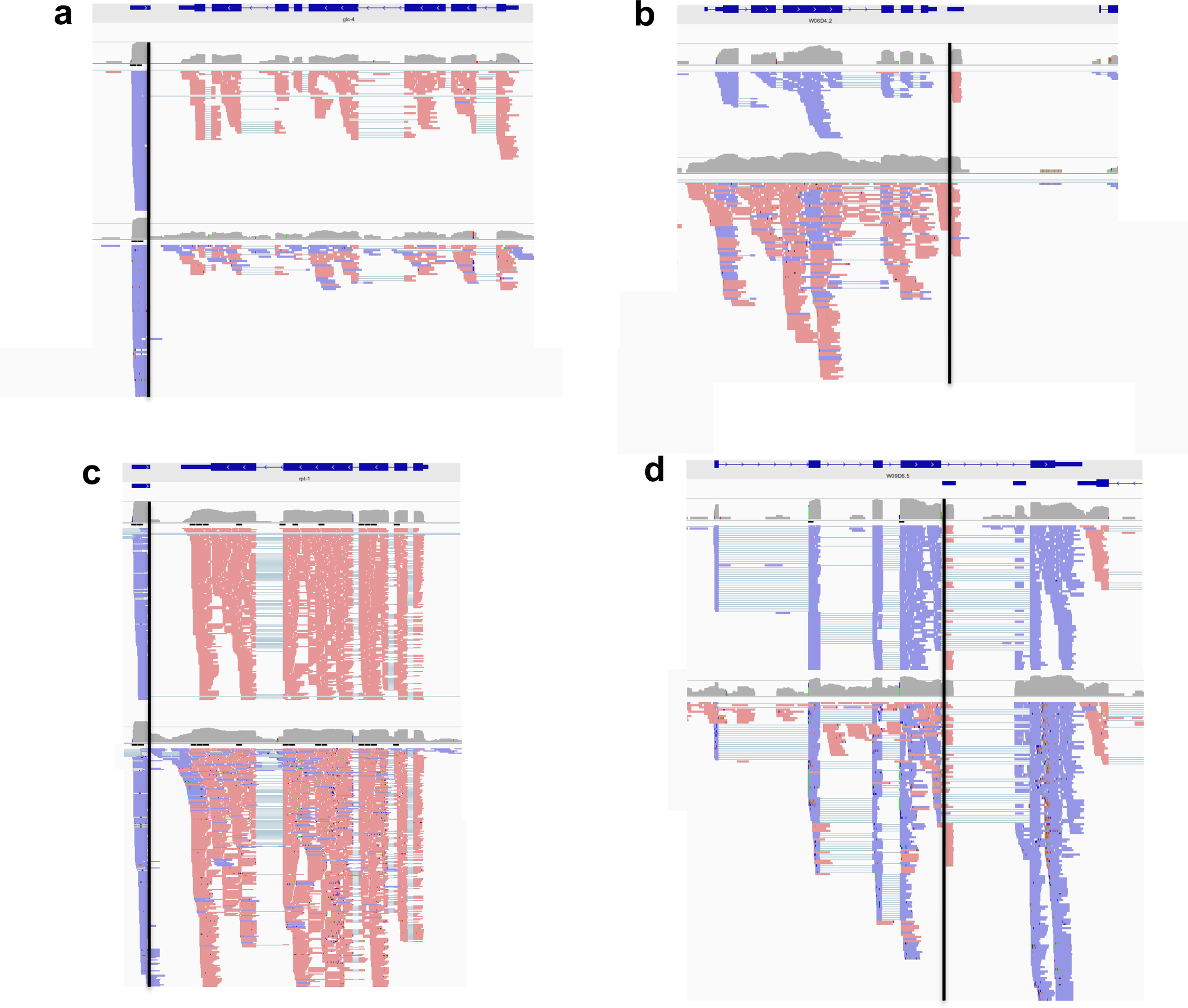

Supplement: S13 Fig — Directional RNAseq alignments of WT and ints-6 (t1903) mutant worms to the C. elegans reference genome. Reads on the + strand are shown in blue and reads on the–strand are shown in red. The black line marks the 3’ end of the snRNA. For each case, the upper track shows the genomic region of snRNA loci located downstream and opposite to coding genes. The middle track shows the RNAseq alignment of WT worms. RNAseq shows only the mRNA and the mature snRNA. The lower track shows the RNAseq alignment of ints-6 (t1903) mutant worms. Both types of transcripts are present: mRNA and antisense RNAs on the opposite strand, derived from the lack of processing of snRNAs located in antisense downstream of the gene. (a) Shows the gene glc-4, (b) the gene W06D4.2, (c) the gene rpt-1 and (d) the gene W09D6.5. (TIF) [file pgen.1007981.s013.tif]

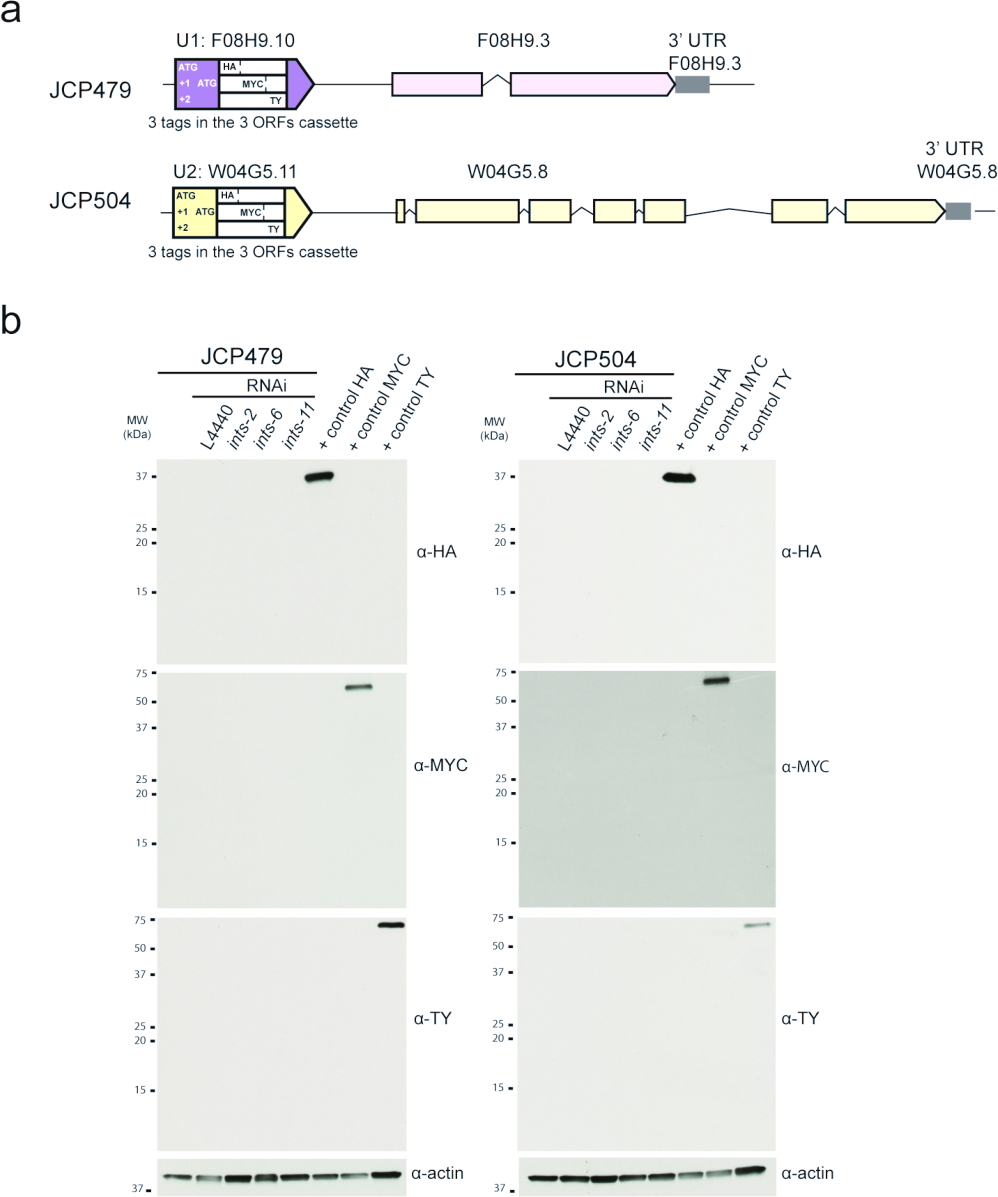

Supplement: S14 Fig — (a) Scheme of the plasmids made and integrated into chromosome II, using the mosSCI system. Plasmid pJC63 was used to generate the JCP479 transgenic strain. A genomic region amplified upstream of U1 F08H9.10 to the downstream region of the F08H9.3 gene is shown. A cassette with the HA, MYC (+1b) and TY (+2b) tags in each of the 3 ORFs was inserted into U1 F08H9.3 so that the HA tag and the MYC tag were in-frame respectively with the first and the second ATGs of the U1 F08H9.10. Plasmid pJC64 was used to generate the JCP504 strain. A genomic region amplified upstream of U2 W04G5.11 to the downstream region of the W04G5.8 gene is shown. The same cassette with the HA, MYC (+1b) and TY (+2b) tags in each of the 3 ORFs was inserted into the U2 W04G5.11. (b) WBs of protein extracts from the JCP479 and JCP504 transgenic strains after RNAi treatment of the C. elegans Integrator complex subunits ints-2, -9 and -11 and the empty L4440 vector. Expected molecular weights: JCP479/JCP504 for HA (1st ORF): 7 kDa; MYC (2nd ORF): 8.7 kDa; TY (3rd ORF): 5.5 kDa. Positive control HA: 28.4 kDa; positive control MYC: 61.5 kDa; positive control TY: 54.3 kDa. (TIF) [file pgen.1007981.s014.tif]

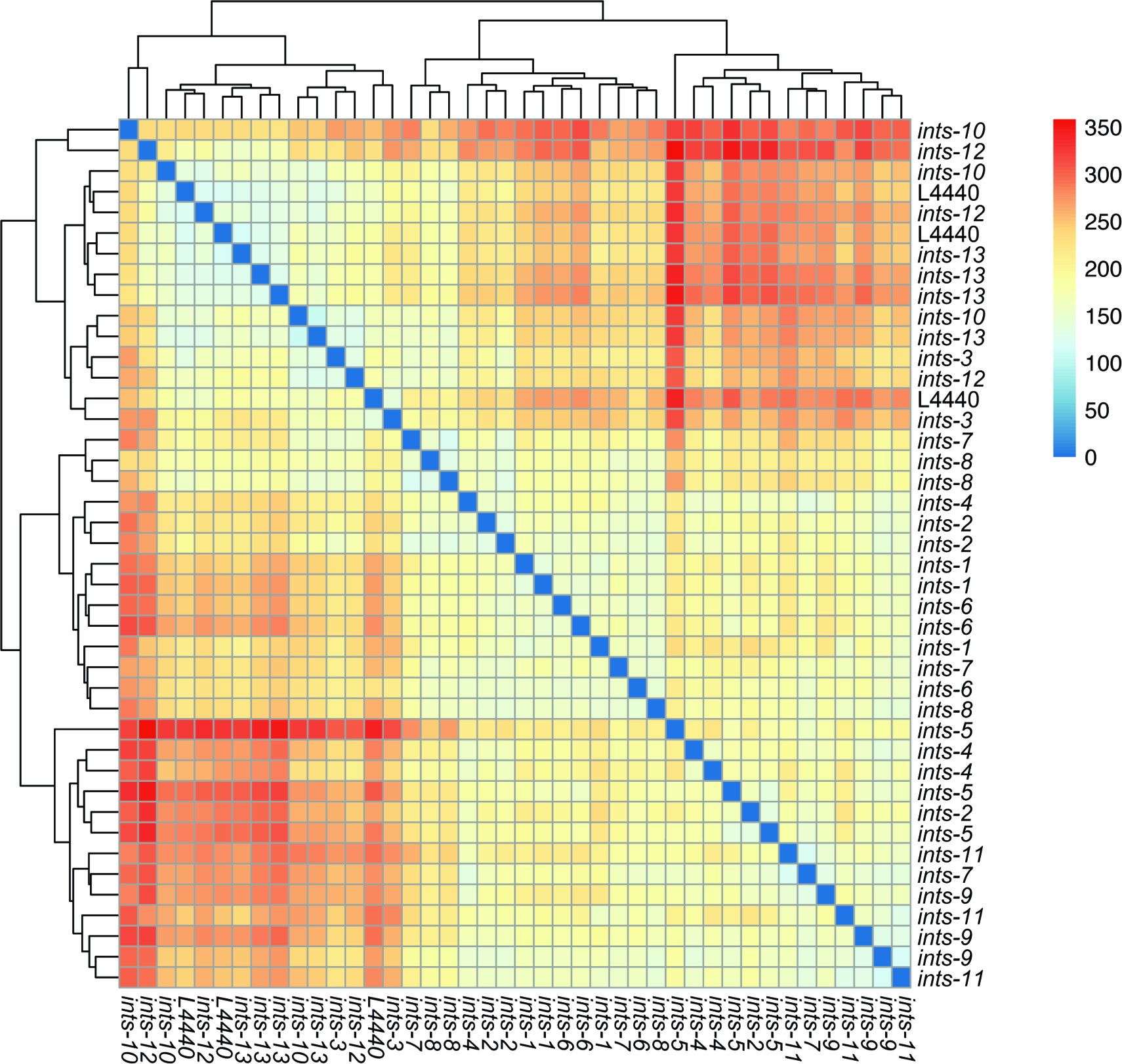

Supplement: S15 Fig — Sample-to-sample distance heatmap showing the Euclidean distances (calculated from the rld data) between worm samples. Upper and left-side dendrograms show samples grouped by similarity of their transcriptional profiles. (TIF) [file pgen.1007981.s015.tif]
