# Supplementary material for: Disruption of the Caenorhabditis elegans Integrator complex triggers a non-conventional transcriptional mechanism beyond snRNA genes
Source: PLoS Genet. 2019 Feb 26;15(2):e1007981. doi: 10.1371/journal.pgen.1007981 (PMC6390993; doi:10.1371/journal.pgen.1007981)
Supplement: S3 Table — Sequence of the different snRNAs in C. elegans. CLUSTAL multiple sequence alignment by MUSCLE (3.8) and consensus sequence of each snRNA type. (DOC) [file pgen.1007981.s018.doc]

**snRNA U1**

sup-6 -AAACTTACCTGGCTGGGGGTTATTTCGTGATCATGAAGACGGAATCCCCATGGTGAGGC

sup-39 TAAACTTACCTGGCTGGGGG-TATCTCGTGATCATGAAGACGGGATCCCCATGGTGAGGC

C27H5.11 -AAACTTACCTGGCTGGGGGCTATTTTGCGATCAAGAAGGCAGAATCCCCATGGTGAGGC

H27M09.6 -AAACTTACCTGGCTGGGGGTTATTTCGCGATCACAAAGGCGGAATCCCCATGGTTAGGC

F08H9.11 -AAACTTACCTGGCTGGGGGTTATTTCGCGATCAAGAAGGCGGAATCCCCATGGTGAGGC

F40G12.14 -AAACTTACCTGGCTGGGGGTTATTTCGCGATCAAGAAGGCGGAATCCCCATGGTGAGGC

F08H9.10 -AAACTTACCTGGCTGGGGGTTATTTCGCGATCAAGAAGGCGGAATCCCCATGGTGAGGC

T08G5.11 -AAACTTACCTGGCTGGGGGTTATTTCGCGATCAAGAAGGCGGAATCCCCATGGTGAGGC

F40G12.13 -AAACTTACCTGGCTGGGGGTTATTTCGCGATCAAGAAGGCGGAATCCCCATGGTGAGGC

T27E4.12 -AAACTTACCTGGCTGGGGGTTATCTCGCGATCATGAAGGCGGGATCCCCATGGTGAGGC

T27E4.11 -AAACTTACCTGGCTGGGGGTTATCTCGCGATCATGAAGGCGGGATCCCCATGGTGAGGC

T27E4.10 -AAACTTACCTGGCTGGGGGTTATCTCGCGATCATGAAGGCGGGATCCCCATGGTGAGGC

******************* *** * * ***** *** * * *********** ****

sup-6 CTACCCATTGCACTTTTGG-GCGGGCTGACCCG------TGTGGCAGTCTCGAGTTGAGA

sup-39 CTACCCATTGCACTTTTGG-GCGGGCTGACCTG------TGTGGCAGTCTCGAGTTGAGA

C27H5.11 CTACCCATTGCACTTTTGG-GCGGGCTGACCTG------TGTGGCAGTCTCGAGTTGAGA

H27M09.6 CTACCCATTGCACTTTTGGTGCGGGCTGACCTG------TGTGGCAGTCTCGAGTTGAGA

F08H9.11 CTACCCATTGCACTTTTGG-GCGGGCTGACCTG------TGTGGCAGTCTCGAGTTGAGA

F40G12.14 CTACCCATTGCACTTTTGG-GCGGGCTGACCTG------TGTGGCAGTCTCGAGTTGAGA

F08H9.10 CTACCCATTGCACTTTTGG-GCGGGCTGACCTA------TGTGGCAGTCTCGAGTTGAGA

T08G5.11 CTACCCATTGCACTTTTGG-GCGGGCTGACCTG------TGTGGCAGTCTCGAGTTGAGA

F40G12.13 CTACCCATTGCACTTTTGG-GCGGGCTGACCTG------TGTGGCAGTCTCGAGTTGAGA

T27E4.12 CTATCCATTGCACTTTTGG-ATGGGCTGACTCGCAGTCTCAGGGCAGTCTCGAGTTGAGA

T27E4.11 CTATCCATTGCACTTTTGG-ATGGGCTGACCTG------TGTGGCAGTCTCGAGTTGAGA

T27E4.10 CTATCCATTGCACTTTTGG-ATGGGCTGACCTG------TGTGGCAGTCTCGAGTTGAGA

*** *************** ******** ******************

sup-6 TTCGCCAACAGCTTAATTTTTGCGTATCGGGGCTGCGTGCGCGCGGCCCTGAA

sup-39 TTCGCCAACAGCTTAATTTTTGCGTATCGGGGCTGCGTGCGCGCGGCCCTGAA

C27H5.11 TTCGCCAACAGCTTAATTTTTGCGTATCGGGGCTGCGTACGCGCGGCCCTG--

H27M09.6 TTCGCCAACAGCTTAATTTTTGCGTATCGGGGCTGCGTGCGCGCGGCCCTGA-

F08H9.11 TTCGCCAACAGCTTAATTTTTGCGTATCGGGGCTGCGTGCGCGCGGCCCTAAA

F40G12.14 TTCGCCAACAGCTTAATTTTTGCGTATCGGGGCTGCGTGCGCGCGGCCCTGTA

F08H9.10 TTCGCCAACAGCTTAATTTTTGCGTATCGGGGCTGCGTGCGCGCGGCCCTGAA

T08G5.11 TTCGCCAACAGCTTTATTTTTGCGTATCGGGGCTGCGTGCGCGCGGCCCTGAA

F40G12.13 TTCGCCAACAGCTTAATTTTTGCGTATCGGGGCTGCGTGCGCGCGGCCCTGAA

T27E4.12 TTCGCCAACAGCTTAATTTTTGCGTATCGGGGCTGCGTGCGCGCGGCCCTGA-

T27E4.11 TTCGCCAACAGCTTAATTTTTGCGTATCGGGGCTGCGTGCGCGCGGCCCCGA-

T27E4.10 TTCGCCAACAGCTTAATTTTTGCGTATCGGGGCTGCGTGCGCGCGGCCCTGA-

************** *********************** **********

>Consensus U1

AAACTTACCTGGCTGGGGGTTATTTCGCGATCAAGAAGGCGGAATCCCCATGGTGAGGCCTACCCATTGCACTTTTGGGCGGGCTGACCTGTGTGGCAGTCTCGAGTTGAGATTCGCCAACAGCTTAATTTTTGCGTATCGGGGCTGCGTGCGCGCGGCCCTGAA

**snRNA U2**

T14B4.10 ATCGCTTCTTCGGCTTATTAGCTAATATCAAAGTGTAG--TCTGTTCTCATCGTACTAAC

F11A5.14 ATCGCTTCTTCGGCTTATTAGCTAAGATCAAAGTGTAGTATCTGTTCTTATCGTATTAAC

C05G6.4 -TCGCTTCTTCGGCTTATTAGCTAAGATCAAAGTGTAGTATCTGTTCTTATCGTATTAAC

W04G5.11 ATCGCTTCTTCGGCTTATTAGCTAAGATCAAAGTGTAGTATCTGTTCTTATCGTATTAAC

F15H9.5 ATCGCTTCTTCGGCTTATTAGCTAAGATCAAAGTGTAGTATCTGTTCTTATCGTATTAAC

F15H9.6 ATCGCTTCTTCGGCTTATTAGCTAAGATCAAAGTGTAGTATCTGTTCTTATCGTATTAAC

Y47H9A.2 ATCGCTTCTTCGGCTTATTAGCTAAGATCAAAGTGTAGTATCTGTTCTTATCGTATTAAC

F56H6.15 ATCGCTTCTTCGGCTTATTAGCTAAGATCAAAGTGTAGTATCTGTTCTTATCGTATTAAC

Y54G9A.8 ATCGCTTCTTCGGCTTATTAGCTAAGATCAAAGTGTAGTATCTGTTCTTATCGTATTAAC

F56H6.16 ATCGCTTCTTCGGCTTATTAGCTAAGATCAAAGTGTAGTATCTGTTCTTATCGTATTAAC

C47F8.9 ATCGCTTCTTCGGCTTATTAGCTAAGATCAAAGTGTAGTATCTGTTCTTATCGTATTAAC

F56H6.14 ATCGCTTCTTCGGCTTATTAGCTAAGATCAAAGTGTAGTATCTGTTCTTATCGTATTAAC

R05D7.6 ATCGCTTCTTCGGCTTATTAGCTAAGATCAAAGTGTAGTATCTGTTCTTATCGTATTAAC

F08G2.10 ATCGCTTCTTCGGCTTATTAGCTAAGATCAAAGTGTAGTATCTGTTCTTATCGTATTAAC

W07G1.9 ATCGCTTCTTCGGCTTATTAGCTAAGATCAAAGTGTAGTATCTGTTCTTATCGTATTAAC

F08G2.11 ATCGCTTCTTCGGCTTATTAGCTAAGATCAAAGTGTAGTATCTGTTCTTATCGTATTAAC

F08G2.9 ATCGCTTCTTCGGCTTATTAGCTAAGATCAAAGTGTAGTATCTGTTCTTATCGTATTAAC

W07G1.8 ATCGCTTCTTCGGCTTATTAGCTAAGATCAAAGTGTAGTATCTGTTCTTATCGTATTAAC

************************ ************ ******** ****** ****

T14B4.10 CTACGGTATACAGTCGAATGGGCGTAATAAAGGTTATATGATTTTTGGAACCTAGGGAAG

F11A5.14 CTACGGTATACACTCGAATGAGTGTAATAAAGGTTATATGATTTTTGGATCCTAGGAAAG

C05G6.4 CTACGGTATACACTCAAATGAGTGTAATAAAGGTTATATGATTTTTGGAACCCTGGGAAG

W04G5.11 CTACGGTATACACTCGAATGAGTGTAATAAAGGTTATATGATTTTTGGAACCCAGGGAAG

F15H9.5 CTACGGTATACACTCGAATGAGTGTAATAAAGGTTATAAGATTTTTGGAACCTAGGGAAG

F15H9.6 CTACGGTATACACTCGAATGAGTGTAATAAAGGTTATAAGATTTTTGGAACCTAGGGAAG

Y47H9A.2 CTACGGTATACACTCGAATGAGTGTAATAGAGGTTATATGATTTTTGGAACCTAGGGAAG

F56H6.15 CTACGGTATACACTCGAATGAGTGTAATAAAGGTTATATGATTTTTGGAACCTAGGGAAG

Y54G9A.8 CTACGGTATACACTCGAATGAGTGTAATAAAGGTTATATGATTTTTGGAACCTAGGGAAG

F56H6.16 CTACGGTATACACTCGAATGAGTGTAATAAAGGTTATATGATTTTTGGAACCTAGGGAAG

C47F8.9 CTACGGTATACACTCGAATGAGTGTAATAAAGGTTATATGATTTTTGGAACCTAGGGAAG

F56H6.14 CTACGGTATACACTCGAATGAGTGTAATAAAGGTTATATGATTTTTGGAACCTAGGGAAG

R05D7.6 CTACGGTATACACTCGAATGAGTGTAATAAAGGTTATATGATTTTTGGAACCTAGGGAAG

F08G2.10 CTACGGTATACACTCGAATGAGTGTAATAAAGGTTATATGATTTTTGGAACCTAGGGAAG

W07G1.9 CTACGGTATACACTCGAATGAGTGTAATAAAGGTTATATGATTTTTGGAACCTAGGGAAG

F08G2.11 CTACGGTATACACTCGAATGAGTGTAATAAAGGTTATATGATTTTTGGAACCTAGGGAAG

F08G2.9 CTACGGTATACACTCGAATGAGTGTAATAAAGGTTATATGATTTTTGGAACCTAGGGAAG

W07G1.8 CTACGGTATACACTCGAATGAGTGTAATAAAGGTTATATGATTTTTGGAACCTAGGGAAG

************ ** **** * ****** ******** ********** ** ** ***

T14B4.10 ACTCGGGGCTTTCTCCGACTTCCCAAGGGTCGTCCTGGCATTTCACTGCTGCTGGG--CG

F11A5.14 ACTCGGGGCTTGCTCCGACTTTCCGCGGGTCGTACTGGCGTTGCACTGCTGCCGGGCTCG

C05G6.4 ACTCGGGGCTTGCTTCGACTTCCCAAGGGTCGTCCTGGCGTTGCACTTCTGCCGGGCTCG

W04G5.11 ACTCGGGGCTTGCTCCGACTTCCCAAGGGTCGTCCTGGCGTTGCACTGCTGCCGGGCTCG

F15H9.5 ACTCGGGGCTTGCTCCGACTTCCCAAGGGTCGTCCTGGCGTTGCACTGCTGCCGGGCTCG

F15H9.6 ACTCGGGGCTTGCTCCGACTTCCCAAGGGTCGTCCTGGCGTTGCACTGCTGCCGGGCTCG

Y47H9A.2 ACTCGGGGCTTGCTCCGACTTCCCAAGGGTCGTCCTGGCGTTGCACTGCTGCCGGGCTCG

F56H6.15 ACTCGGGGCTTGCTCCGACTTCCCAAGGGTCGTCCTGGCGTTGCACTGCTGTCGGGCTCG

Y54G9A.8 ACTCGGGGCTTGCTCCGACTTCCCAAGAGTCGTCCTGGCGTTGCACTGCTGCCGGGCTCG

F56H6.16 ACTCGGGGCTTGCTCCGACTTCCCAAGGGTCGTCCTGGCGTTGCACTGCTGCCGGGCTCG

C47F8.9 ACTCGGGGCTTGCTCCGACTTCCCAAGGGTCGTCCTGGCGTTGCACTGCTGCCGGGCTCG

F56H6.14 ACTCGGGGCTTGCTCCGACTTCCCAAGGGTCGTCCTGGCGTTGCACTGCTGCCGGGCTCG

R05D7.6 ACTCGGGGCTTGCTCCGACTTCCCAAGGGTCGTCCTGGCGTTGCACTGCTGCCGGGCTCG

F08G2.10 ACTCGGGGCTTGCTCCGACTTCCCAAGGGTCGTCCTGGCGTTGCACTGCTGCCGGGCTCG

W07G1.9 ACTCGGGGCTTGCTCCGACTTCCCAAGGGTCGTCCTGGCGTTGCACTGCTGCCGGGCTCG

F08G2.11 ACTCGGGGCTTGCTCCGACTTCCCAAGGGTCGTCCTGGCGTTGCACTGCTGCCGGGCTCG

F08G2.9 ACTCGGGGCTTGCTCCGACTTCCCAAGGGTCGTCCTGGCGTTGCACTGCTGCCGGGCTCG

W07G1.8 ACTCGGGGCTTGCTCCGACTTCCCAAGGGTCGTCCTGGCGTTGCACTGCTGCCGGGCTCG

*********** ** ****** ** * ***** ***** ** **** *** *** **

T14B4.10 GCCCAGTCCC

F11A5.14 GCCCAGT---

C05G6.4 GCCCAGT---

W04G5.11 GCCCA-----

F15H9.5 GCCCAGT---

F15H9.6 GCCCAGT---

Y47H9A.2 GCCCAGT---

F56H6.15 GCCCAGT---

Y54G9A.8 GCCCAGT---

F56H6.16 GCCCAGT---

C47F8.9 GCCCAGT---

F56H6.14 GCCCAGT---

R05D7.6 GCCCAGT---

F08G2.10 GCCCAGT---

W07G1.9 GCCCAGT---

F08G2.11 GCCCAGT---

F08G2.9 GCCCA-----

W07G1.8 GCCCAGT---

*****

>Consensus U2

ATCGCTTCTTCGGCTTATTAGCTAAGATCAAAGTGTAGTATCTGTTCTTATCGTATTAACCTACGGTATACACTCGAATGAGTGTAATAAAGGTTATATGATTTTTGGAACCTAGGGAAGACTCGGGGCTTGCTCCGACTTCCCAAGGGTCGTCCTGGCGTTGCACTGCTGCCGGGCTCGGCCCAGT

**snRNA U4**

F10D2.13 agctttgcgctggggcgataacgtgaccaatgaggctttgccgaggtgcgtttattgctg

T11F9.16 agctttgcgctggggcgataacgtgaccaatgaggctttgccgaggtgcgtttattgctg

C52E4.8 agctttgcgctggggcgataacgtgaccaatgaggctttgccgaggtgcgtttattgctg

T11F9.15 agctttgcgctggggcgataacgtgaccaatgaggctttgccgaggtgcgtttattgctg

K03B8.10 agctttgcgctggggcgataacgtgaccaatgaggctttgccgaggtgcgtttattgctg

************************************************************

F10D2.13 gttgaaaacttttcccaattgcccgcgatgacctctgaaacatgggtgccatacgcaatt

T11F9.16 gttgaaaacttttcccaattgcccgcgatgtcccctgaaacatgggtggcatacgcaatt

C52E4.8 gttgaaaacttttcccaattgcccgcgatgtcccctgaaacatgggtggcatacgcaatt

T11F9.15 gttgaaaacttttcccaattgcccgcgatgtcccctgaaacatgggtggcatacgcaatt

K03B8.10 gttgaaaacttttcccaattgcccgcgatgtcccctgaaacatgggtggcatacgcaatt

****************************** ** ************** ***********

F10D2.13 tttgaacgcctctaggaggcag--

T11F9.16 tttgaaagcctctaggaggcagaa

C52E4.8 tttgaacgcctctaggaggcagaa

T11F9.15 tttgaacgcctctaggaggcagaa

K03B8.10 tttgaacgcctccaggaggcagaa

****** ***** *********

>Consensus U4

AGCTTTGCGCTGGGGCGATAACGTGACCAATGAGGCTTTGCCGAGGTGCGTTTATTGCTGGTTGAAAACTTTTCCCAATTGCCCGCGATGTCCCCTGAAACATGGGTGGCATACGCAATTTTTGAACGCCTCTAGGAGGCAGAA

**snRNA U5**

F32B5.9 CAACTCTGGTTCCTCTGCATTTAACCGTGAAAATCTTTCGCCTTTTACTAAAGATTTCCG

F42C5.12 CAACTCTGGTTCCTCTGCATTTAACCGTGAAAATCTTTCGCCTTTTACTAAAGATTTCCG

C53D6.16 ---CTCTGGTTCCTCTGCATTTAACCGTGAAAATCTTTCGCATTTTACTAAAGATTTCCG

Y69A2AR.36 -AACTCTGGTTCCTCTGCATTTAACCGTGAAAATCTTTCGCCTTTTACTAAAGATTTCCG

C53B4.9 -AACTCTGGTTCCTCTGCATTTAACCATGAAAATCTTTCGCCTTTTACTAAAGATTTCCG

F38E11.11 -AACTCTGGTTCCTCTGCATTTAACCGTGAAAATCTTTCGCCTTTTACTAAAGATTTCCG

F38E11.10 -AACTCTGGTTCCTCTGCATTTAACCGTGAAAATCTTTCGCCTTTTACTAAAGATTTCCG

C53D6.12 -AACTCTGGTTCCTCTGCATTTAACCGTGAAAATCTTTCGCCTTTTACTAAAGATTTCCG

ZK897.3 -AACACTGGTTCCTCTGCATTTAACCGTGAAAATCTTTCGCCTTTTACTAAAGATTTCCG

F07C6.5 -AACTCTGGTTCCTCTGCATTTAACCGTGAAAATCTTTCGCCTTTTACTAAAGATTTCCG

ZK897.2 -AACTCTGGTTCCTCTGCATTTAACCGTGAAAATCTTTCGCCTTTTACTAAAGATTTCCG

ZK897.4 -AACTCTGGTTCCTCTGCATTTAACCGTGAAAATCTTTCGCCTTTTACTAAAGATTTCCG

* ********************* ************** ******************

F32B5.9 TGCAAAGGAGCATACATTGAGTATTACTTAGAATTTTTGGAGCCTTCTCGAAAGAGCAAG

F42C5.12 TGCAAAGGAGCATTTACTGAGTATTACATACAATTTTTGGAGACTCCTTGAGAAAGCGGG

C53D6.16 TGCTAAGGAGCATACATTGAGTATTATATACAATTTTTGGAGT-TCCTTGAGAAAACGAG

Y69A2AR.36 TGCAAAGGAGCATACATTGAGTATTATACACAAATTTTGGAGTCCCCTCGGAAGAGCGGG

C53B4.9 TGCAAAGGAGCATACATTGAGTATTGTATACAATTTTTGGAGTCCCCTTGAGAAAGCGGG

F38E11.11 TGCAAAGGAGCATACATTGAGTATTATATACAATTTTTGGAGTCCCCTTGAGAAAGCGGG

F38E11.10 TGCAAAGGAGCATACATTGAGTATTATATACAATTTTTGGAGTCCCCTTGAGAAAGCGGG

C53D6.12 TGCAAAGGAGCATACATTGAGTATTATATATAATTTTTGGAGTCCCCTTGAGAAAGCGGG

ZK897.3 TGCAAAGGAGCATACGTTGAGTATTATATACAATTTTTGGAGTCCCCTCGAAAGAGCGGG

F07C6.5 TGCAAAGGAGCATACATTGAGTATTATATACAATTTTTGGAGTCCCCTCGAAAGAGCGGG

ZK897.2 TGCAAAGGAGCATACATTGAGTATTATATACAATTTTTGGAGTCCCCTCGAAAGAGCGGG

ZK897.4 TGCAAAGGAGCATACATTGAGTATTATATACAATTTTTGGAGTCCCCTCGAGAGAGCGGG

*** ********* ******** * ** ******** ** * * * * *

F32B5.9 GCA----

F42C5.12 TC-----

C53D6.16 A------

Y69A2AR.36 AC-----

C53B4.9 ACA----

F38E11.11 ACA----

F38E11.10 ACA----

C53D6.12 ACAAAAT

ZK897.3 ACA----

F07C6.5 ACA----

ZK897.2 ACA----

ZK897.4 ACA----

>Consensus U5

NAACTCTGGTTCCTCTGCATTTAACCGTGAAAATCTTTCGCCTTTTACTAAAGATTTCCGTGCAAAGGAGCATACATTGAGTATTATATACAATTTTTGGAGTCCCCTXGAGAXAGCGGGACAXXXX

**snRNA U6**

B0205.15 GCTCTTCCGAGAACATATACTAAAATTGGACTAATACAGAGAAGATTAGCATGGCCCCTG

T20D3.12 ---------AGTATATGTTCTAGAATTGGAACAATACAGAGAAGATTAGCATGGCCCCTG

T20D3.13 -----TTCTAGAACATATACTAAAATTGGAACAATACAGAGAAGATTAGCATGGCCCCTG

F36A4.23 TTTCTTCCGAGAACATATACTAAAATTGGAACAATATAGAGAAGATTAGCATGGCCCCTG

F32D1.12 TTTCTTCCGAGAACATATACTAAAATTGGAACAATATAGAGAAGATTAGCATGGCCCCTG

F54C8.10 GTTCTTCCAAGAACATATACTAAAATTGGAACAATACAGAGAAGATTAGCATGGCCCCTG

R07E5.16 GTTCTTCCGAGAACATATACTAAAATTGGAACAATACAGAGAAGATTAGCATGGCCCCTG

C28A5.7 GTTCTTCCGAGAACATATACTAAAATTGGAACAATACAGAGAAGATTAGCATGGCCCCTG

F54D8.7 GTTCTTCCGAGAACATATACTAAAATTGGAACAATACAGAGAAGATTAGCATGGCCCCTG

F54C8.8 GTTCTTCCGAGAACATATACTAAAATTGGAACAATACAGAGAAGATTAGCATGGCCCCTG

F54C8.9 GTTCTTCCGAGAACATATACTAAAATTGGAACAATACAGAGAAGATTAGCATGGCCCCTG

W05B2.8 GTTCTTCCGAGAACATATACTAAAATTGGAACAATACAGAGAAGATTAGCATGGCCCCTG

Y17G9A.85 GTTCTTCCGAGAACATATACTAAAATTGGAACAATACAGAGAAGATTAGCATGGCCCCTG

Y17G9A.90 GTTCTTCCGAGAACATATACTAAAATTGGAACAATACAGAGAAGATTAGCATGGCCCCTG

T08B6.62 GTTCTTCCGAGAACATATACTAAAATTGGAACAATACAGAGAAGATTAGCATGGCCCCTG

Y9C9A.111 GTTCTTCCGAGAACATATACTAAAATTGGAACAATACAGAGAAGATTAGCATGGCCCCTG

K09B11.12 GTTCTTCCGAGAACATATACTAAAATTGGAACAATACAGAGAAGATTAGCATGGCCCCTG

K09B11.13 GTTCTTCCGAGAACATATACTAAAATTGGAACAATACAGAGAAGATTAGCATGGCCCCTG

K09B11.14 GTTCTTCCGAGAACATATACTAAAATTGGAACAATACAGAGAAGATTAGCATGGCCCCTG

K09B11.15 GTTCTTCCGAGAACATATACTAAAATTGGAACAATACAGAGAAGATTAGCATGGCCCCTG

K09B11.16 GTTCTTCCGAGAACATATACTAAAATTGGAACAATACAGAGAAGATTAGCATGGCCCCTG

K09C6.12 GTTCTTCCGAGAACATATACTAAAATTGGAACAATACAGAGAAGATTAGCATGGCCCCTG

K09B11.11 GTTCTTCCGAGAACATATACTAAAATTGGAACAATACAGAGAAGATTAGCATGGCCCCTG

** * ** * *** ******* **** ***********************

B0205.15 CGCAAGGATAACACGCAAA---AGTAGACGTTCCAACCGACACGCCTCTTT

T20D3.12 CGCACGGATGACACGCAAATTCGTGAAGCGTTCCAA---------ATTTTT

T20D3.13 CGCACGGATGACACGCAAATTCGTGAAGCGTTCCAA---------ATTTTT

F36A4.23 CGCAAGGATGACACGCAAATTCGTGAAGCGTTCCAA---------ATTTTT

F32D1.12 CGCAAGGATGACACGCAAATTCGTGAAGCGTTCCAA---------ATTTTT

F54C8.10 CGCAAGGATGACACGCAAATTCGTGAAGCGTTCCAA---------ATTTTT

R07E5.16 CGCAAGGATGACACGCAAATTCGTGAAGCGTTCCAA---------ATTTTT

C28A5.7 CGCAAGGATGACACGCAAATTCGTGAAGCGTTCCAA---------ATTTTT

F54D8.7 CGCAAGGATGACACGCAAATTCGTGAAGCGTTCCAA---------ATTTT-

F54C8.8 CGCAAGGATGACACGCAAATTCGTGAAGCGTTCCAA---------ATTTTT

F54C8.9 CGCAAGGATGACACGCAAATTCGTGAAGCGTTCCAA---------ATTTTT

W05B2.8 CGCAAGGATGACACGCAAATTCGTGAAGCGTTCCAA---------ATTTTT

Y17G9A.85 CGCAAGGATGACACGCAAATTCGTGAAGCGTTCCAA---------ATTTT-

Y17G9A.90 CGCAAGGATGACACGCAAATTCGTGAAGCGTTCCAA---------ATTTTT

T08B6.62 CGCAAGGATGACACGCAAATTCGTGAAGCGTTCCAA---------ATTTT-

Y9C9A.111 CGCAAGGATGACACGCAAATTCGTGAAGCGTTCCAA---------ATTTTT

K09B11.12 CGCAAGGATGACACGCAAATTCGTGAAGCGTTCCAA---------ATTTTT

K09B11.13 CGCAAGGATGACACGCAAATTCGTGAAGCGTTCCAA---------ATTTTT

K09B11.14 CGCAAGGATGACACGCAAATTCGTGAAGCGTTCCAA---------ATTTTT

K09B11.15 CGCAAGGATGACACGCAAATTCGTGAAGCGTTCCAA---------ATTTTT

K09B11.16 CGCAAGGATGACACGCAAATTCGTGAAGCGTTCCAA---------ATTTTT

K09C6.12 CGCAAGGATGACACGCAAATTCGTGAAGCGTTCCAA---------ATTTTT

K09B11.11 CGCAAGGATGACACGC-AATTCGTGAAGCGTTCCAA---------ATTTTT

**** **** ****** ** * ******** * **

>Consensus U6

GTTCTTCCGAGAACATATACTAAAATTGGAACAATACAGAGAAGATTAGCATGGCCCCTGCGCAAGGATGACACGCAAANNTTCGTGNNNNAAGCGTTCCAAATTTTT

**SL**

sls-1.1 ------------------------------TTGAAACTGACCCAAAGAAATTTGGCGTTA

sls-1.2 CGGTTTAATTACCCAAGTTTGAGGTAAACATTGAAACTGACCCAAAGAAATTTGGCGTTA

sls-1.3 CGGTTTAATTACCCAAGTTTGAGGTAAACATTGAAACTGACCCAAAGAAATTTGGCGTTA

sls-1.4 CGGTTTAATTACCCAAGTTTGAGGTAAACATTGAAACTGACCCAAAGAAATTTGGCGTTA

sls-1.5 ------------------------------TTGAAACTGACCCAAAGAAATTTGGCGTTA

sls-1.6 CGGTTTAATTACCCAAGTTTGAGGTAAACATTGAAACTGACCCAAAGAAATTTGGCGTTA

sls-1.7 CGGTTTAATTACCCAAGTTTGAGGTAAACATTGAAACTGACCCAAAGAAATTTGGCGTTA

sls-1.8 CGGTTTAATTACCCAAGTTTGAGGTAAACATTGAAACTGACCCAAAGAAATTTGGCGTTA

sls-1.9 CGGTTTAATTACCCAAGTTTGAGGTAAACATTGAAACTGACCCAAAGAAATTTGGCGTTA

sls-1.10 CGGTTTAATTACCCAAGTTTGAGGTAAACATTGAAACTGACCCAAAGAAATTTGGCGTTA

sls-1.11 CGGTTTAATTACCCAAGTTTGAGGTAAACATTGAAACTGACCCAAAGAAATTTGGCGTTA

sls-1.12 CGGTTTAATTACCCAAGTTTGAGGTAAACATTGAAACTGACCCAAAGAAATTTGGCGTTA

******************************

sls-1.1 GCTATAAATTTTGGAACGTCTCCTCTCGGGGAGACAAA

sls-1.2 GCTATAAATTTTGGAACGTCTCCTCTCGGGGAGACAAA

sls-1.3 GCTATAAATTTTGGAACGTCTCCTCTCGGGGAGACAAA

sls-1.4 GCTATAAATTTTGGAACGTCTCCTCTCGGGGAGACAAA

sls-1.5 GCTATAAATTTTGGAACGTCTCCTCTCGGGGAGACAAA

sls-1.6 GCTATAAATTTTGGAACGTCTCCTCTCGGGGAGACAAA

sls-1.7 GCTATAAATTTTGGAACGTCTCCTCTCGGGGAGACAAA

sls-1.8 GCTATAAATTTTGGAACGTCTCCTCTCGGGGAGACAAA

sls-1.9 GCTATAAATTTTGGAACGTCTCCTCTCGGGGAGACAAA

sls-1.10 GCTATAAATTTTGGAACGTCTCCTCTCGGGGAGACAAA

sls-1.11 GCTATAAATTTTGGAACGTCTCCTCTCGGGGAGACAAA

sls-1.12 GCTATAAATTTTGGAACGTCTCCTCTCGGGGAGACAAA

**************************************

>Consensus SL-1

CGGTTTAATTACCCAAGTTTGAGGTAAACATTGAAACTGACCCAAAGAAATTTGGCGTTAGCTATAAATTTTGGAACGTCTCCTCTCGGGGAGACAAA

sls-2.10 GGTTTAAAACCCAGTTA-CCAAGGTAATTCGGAG-TTCTGACCTTTCGAAAGAAAGCGTC

sls-2.11 GGTTTAAAACCCAGTTA-CCAAGGTAATTCGGAG-TTCTGACCTTTCGAAAGAAAGCGTC

sls-2.9 GGTTTTAACCC-AGTTA-CCAAGGTAATTCGGAG-TTTCGATCTTTCGAAAGAGAGTGTC

sls-2.1 GGTTTTAACCC-AGTTACTCAAGGTACGCTGGAG-TTCTGACCTTTCGAAAGAGAGTGTC

sls-2.4 GGTTTTAACCC-AGTTACTCAAGGTACGCTGGAG-TTCTGACCTTTCGAAAGAAAGTGTC

sls-2.2 GGTTTTAACCC-AGTTACTCAAGGTACGCTGGAG-TTCTGACCTTTCGAAAGAGAGTGTC

sls-2.3 GGTTTTAACCC-AGTTACTCAAGGTACGCTGGAG-TTCTGACCTTTCGAAAGAGAGTGTC

sls-2.8 GGTTTTAACCCAGTTTAACCAAGGTTAGCTGTCG-TTTCGATCTCTCGAGAGAGTGTGTC

sls-2.16 GGTTTTAACCCAAGTTAACCAAGGTTAGCATGGA-ATTCGATCTTTCGAAAGAATGTGTC

sls-2.5 GGTTTTAACCC-AGTTAACCAAGGTTAGCATGGA-ATTCGATCATTCGCAAGAATGTGTC

sls-2.18 GGTTTTAACCC-ATATAACCAAGGTTAGTATGGA-ATTCGATCATTCGCAAGAGTGTGTC

sls-2.14 GGTTTTTACCC-AGTTAACCAAGGTTAGCATTAAATTTCGACCTTTCGCAAGAACGCGTT

sls-2.15 GGTTTATACCC-AGTTAACCAAGGTTAGCATTAAGTTTCGACCTTTC-CAAGAATGTGTT

sls-2.17 GGTTTTAACC--AGTTAACTAAGGTTAACATTAA-TTTCGACCATTCGAAAGATTGTGTT

sls-2.12 GGTTTTAACCC-AGTTAATTGAGGTTAGCAATAA-TTTCGACCTTTCGAAAGATTGTGTT

sls-2.13 GGTTTTAACCC-AGTTAATTGAGGTTAGCAATAA-TTTCGACCTTTCGAAAGATTGTGTT

sls-2.6 GGTTTTAACCC-AGTTAACCAAGGTTAGAATGGA-TTCCGACCATTCGAAAGAGTGTGTT

sls-2.7 GGTTTTAACCC-AGTTAACCAAGGTTAGCATGTA-TTCCGACCATTCGTAAGAGTGTGTT

***** * * ** **** * ** * ** *** * **

sls-2.10 T-TTTACAA--TAAATTTTGGATTAGTTCAGTCGGGGT-TTCCGGCTGAACAAAA---

sls-2.11 T-TTTACGA--TAAATTTTGGATTAGTTCAGTCGGGGT-TTCCGGCTGAACAAAA---

sls-2.9 GATTGTGAA--CAATTTTTGGAATAGCTCTTCCGGGGA-ATCCGGTCGGGCAATA---

sls-2.1 AAACAACTT--TAATTTTTGGAAAAGCTTCGCTGGGGTTATCCGGCGAAGCAAA----

sls-2.4 AAACGACTT--TAATTTTTGGAACCGCTCTGCTGGGGTCATCCGGTAGAGCAAA----

sls-2.2 AAACAACTT--TAACTTTTGGAACCGCTCTGCTGGGGTTATCCGGTAGAGCAAA----

sls-2.3 AAACAACTT--TAATTTTTGGAACTGCTCTACTGGGGTTATCCGGTAGAGCAAA----

sls-2.8 GAATAAAAAA-TAATTTTTGGAATCGCTTCATCGGGGA-ATCCGTTGAAGCAAA----

sls-2.16 GAA-CACAA--AAATTTTTGGAACAGCTTCCTCGGGGT-TTCCGTGGAGGCAAA----

sls-2.5 GAAACACAA----AATTTTGGACAAGCTTCCTCGGGGT-ATCCGTGGGAGCAAA----

sls-2.18 GAA-AACAC--CAAATTTT-GACAAGCTTCTTCGGGGT-ATCCGTGGGAGCAAA----

sls-2.14 GAAATGCAAATCAATTTTTGGAACCGCTTCTTCGGGGA-ATCCGTTGAGGCAAA----

sls-2.15 GAAATGCAAATTAATTTTTGGAACCGCTTCTTCGGGGA-ATCCGTTGAAGCAAAA---

sls-2.17 GAATAACAA--TAATTTTTGGAACAGTTTCTTCGGGGATATCCGATGAAGCAAA----

sls-2.12 GAATAACAA--TAATTTTTGGAACAGCTTCTTCGGGGATATCCGATGAAGCAAA----

sls-2.13 GAATGACAA--TAATTTTTGGAACGACTCCTTCGGGGATATCCGATGAAGCAAA----

sls-2.6 GAATAACAA--TAATTTTTGGAACAGCTTATTCAGGGTTATCCGCAAAAGATAAGAAT

sls-2.7 GAATAACAA--TAATTTTTGGAACAGCTTCTTCGGGGTTATCCGTCGAAGCAAA----

* **** ** * *** **** *

>Consensus SL-2

GGTTTTAACCCAGNTTAACCAAGGTTAGCATGNAATTCCGACCTTTCGAAAGAGTGTGTCGAANTANACAATAATTTTTGGAACAGCTTCTTCGGGGTTATCCGXTGAAGNCAAAXXX

smy-7 -------------------------TACCCTGACCTTG-AAAGGACAGGGTTTGCAAAAA

smy-1 ----------GTTAAACCATAACTAACTCAGGGCCTTGTTAAAGACCCGAAGTACAATTT

smy-12 ----------AATATAATAAAACACTGTCAGGGCCTTACAAAAGACCCACTGATCAATTT

smy-11 -----------ATATAATATATCACTGTCAGGGCCTTACAAAAGACCCAGTGAACAATTT

smy-4 -----------ATATGATACAACACTCTCAGGGCCTGACGAAAGACCCAGTGAACAATTT

smy-6 --------AGCATATGATATAACACTCTCAGGGCCTTACAAAAGACCCAGTGAACTATTT

smy-5 -------------GTATGATATAACACTCAGGGCCTTACAAAAGACCCAGTGAACAATTT

smy-10 CAATCCTAAACTTAAATAACAAAAAACCCAAAGCCTAACTCAGGACTTGGT-AACAATTT

smy-9 ---------------TTCGCTAAACCTCCAAGGCCTTTCATAAGACCTAGTGAACAATTT

smy-8 -----------GTTAATTCATTAACTTCCAAGGCCTTACACAAGACCTAGTGGACAATTT

* *** * *** * *

smy-7 TTGTTCACTGGGTCTTTTGTAAGGCCCTGAGAGTGTTAAATCGTGCTG

smy-1 TTGAAG-CCCTGTCTCT--CGAGGCAGGGG------------------

smy-12 TTGAAAACCTTGTTCTCT-TGAGGGCACGGT-----------------

smy-11 TTGCAAACCCTGTCTTCT-TGAAGGCGGGG------------------

smy-4 TTGAAAACCCTGTCCTTT-CGAGGTCATGGTAA---------------

smy-6 TTGAAAACCCTGTCCTCT-CGAGGTCAGGGTA----------------

smy-5 TTGCAAACCCTGTCCTTT-CGAGGTCAGGGT-----------------

smy-10 TTGGAGACCCTAACTTTTATTAGTTAGGGTG-----------------

smy-9 TTGGGGACCCTATCTTTT-TAAGGTAGGGTTAA---------------

smy-8 TTGGAGACCCTATCTAGC-CGTGATAGGGTG-----------------

*** * *

>Consensus SMY

NNAXXATANNNTXATATAACACTNNNCTCAGGGCCTTACAAAAGACCCAGTGAACAATTTTTGAAAACCCTGTCXTTTCGAGGTCXGGGTXX
